# Association study between genetic variants in retinol metabolism pathway genes and prostate cancer risk

**DOI:** 10.1002/cam4.3538

**Published:** 2020-10-17

**Authors:** Dongliang Cao, Yixuan Meng, Shuwei Li, Junyi Xin, Shuai Ben, Yifei Cheng, Meilin Wang, Lixin Hua, Gong Cheng

**Affiliations:** ^1^ Department of Urology The First Affiliated Hospital of Nanjing Medical University Nanjing Jiangsu People’s Republic of China; ^2^ Department of Genetic Toxicology The Key Laboratory of Modern Toxicology of Ministry of Education School of Public Health Nanjing Medical University Nanjing Jiangsu People’s Republic of China

**Keywords:** genetic variants, prostate cancer, retinol, risk

## Abstract

**Background:**

Evidence suggests that serum retinol level is associated with prostate cancer risk, but the association between genetic variants in the retinol metabolism pathway genes and prostate cancer risk remains unclarified.

**Methods:**

Single‐nucleotide polymorphisms (SNPs) in 31 genes in the retinol metabolism pathway were genotyped to evaluate the association with prostate cancer risk in 4,662 cases and 3,114 controls from the Prostate, Lung, Colorectal and Ovarian (PLCO) Cancer Screening Trial. The gene expression analysis was evaluated using data from the Gene Expression Omnibus (GEO) datasets and the Cancer Genome Atlas (TCGA) database. Data from the Genotype‐Tissue Expression (GTEx) project dataset were utilized to perform the expression quantitative trait loci (eQTL) analysis.

**Results:**

Two SNPs were significantly associated with prostate cancer risk [rs1330286 in *ALDH1A1*: odds ratio (OR) = 0.88, 95% confidence interval (CI) = 0.83‐0.94, *p* = 2.45 × 10^−4^; rs4646653 in *ALDH1A3*: OR = 1.17, 95% CI =1.07‐1.27, *p* = 4.33 × 10^−4^]. Moreover, the mRNA level of *ALDH1A3* was significantly higher in prostate cancer tissues than in normal tissues in both TCGA datasets and GEO datasets (*p* = 1.63 × 10^−12^ and *p* = 4.33 × 10^−2^, respectively). rs1330286 was an eQTL of *ALDH1A1* (*P* = 2.90 × 10^−3^).

**Conclusion:**

Our findings highlight that genetic variants in retinol metabolism pathway genes are associated with prostate cancer risk.

## Graphical abstract

By analyzing data from PLCO database, the genes ALDH1A1 and ALDH1A3 were discovered related to prostate cancer risk.

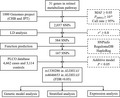

## 1 INTRODUCTION

Among men worldwide, prostate cancer (PCa) ranks second in the incidence rate and is the fifth leading cause of cancer‐related death.^1^ In China, with the development of the economy and changes in lifestyle, an increased incidence trend of prostate cancer was observed from 2000 to 2011.^2^ The natural development of prostate cancer results from numerous risk factors including smoking status, body mass index (BMI), and family history.^3^ Some studies also found that genetic variations play a vital part in the tumorigenesis of prostate cancer and may affect the prognosis as well.^4^

Retinol (vitamin A) is a lipid‐soluble vitamin that is rich in animal liver and green vegetables. It is absorbed in the small intestine by intestinal epithelial cells (IECs).^5^ When retinol is released to circulation by IECs, it combines with retinol‐binding proteins (RBPs) which uptake by target cells with specific receptors on the cell membrane.^6^ After the oxidation reaction in cytoplasm, it binds to retinoic acid receptors (RARs) and retinoid X receptors (RXRs), which are known as two nuclear retinoid receptors, thus active the gene transcription control. As an antioxidant micronutrient, the potential cancer prevention of retinol has raised the interest of many researchers. Published studies have shown that retinol can regulate cell growth, differentiation, and apoptosis by regulating DNA transcription or by interfering with other antioxidants.^7^ Meanwhile, several previous studies reveal that single‐nucleotide polymorphisms (SNPs) in retinol metabolism pathway genes are involved in the development of breast cancer^8^ and pancreatic cancer.^9^ Therefore, the relationship between retinol and prostate cancer risk is still inconsistent, and no association was found between the genetic variation of the retinol metabolic pathway genes and the risk of prostate cancer.

A brief description of retinol metabolism pathway genes enrolled in this study has been proposed as the following: First, serum retinol is taken up by retinol‐binding protein 4 (*RBP4*). The afterward intracellular oxidation of retinol contains two sequential reactions, which are catalyzed by dehydrogenase/reductases (*DHRS3* and *DHRS9*), retinol dehydrogenases (*RDH*s, including *RDH5*, *RDH8*, *RDH10*, *RDH11*, *RDH12*, *RDH13*, *RDH14*, and *RDH16*), alcohol dehydrogenases (*ADH*s, including *ADH4* and *ADH7*) and aldehyde dehydrogenases (*ALDH*s, including *ALDH1A1*, *ALDH1A2*, and *ALDH1A3*).^10^ After that, retinol was oxidized to two isoforms of retinoic acids (RA). There are three RARs in mammals (*RARA*, *RARB* and *RARG*). RARs act in combination with RXRs (*RXRA*, *RXRB*, and *RXRG*) as nuclear retinoid receptors.^11^ RA is transformed into deactivated products by Cytochrome P450 enzymes (*CYP26A1*, *CYP26B1*, and *CYP26C1*).^10^ After searching from online datasets and published studies, β‐carotene oxygenase 1 (*BCO1*), lecithin retinol acyltransferase (*LRAT*), short‐chain dehydrogenase/reductase family 16C member 5 (*SDR16C5*), diacylglycerol O‐acyltransferase 1 (*DGAT1*), aldehyde oxidase 1 (*AOX1*), and retinal pigment epithelium 65 (*RPE65*) were also enrolled in this study. Nancy E Moran et al reported that the genetic variants of *BCO1* are associated with the responses to dietary lycopene intake in prostate tissue.^12^ It is observed that compared to benign prostate tissues, *LRAT* shows a lack of expression in prostate cancer cells.^13^ The hypermethylation of *AOX1* is highly cancer‐specific, making it a promising diagnostic marker of prostate cancer.^14^ Ranjana Mitra and colleagues identified that the inhibition of *DGAT1* leads to prostate cancer cell death.^15^

In this present study, we explored the association between the SNPs of the selected 31 retinol metabolism pathway genes and prostate cancer risk in 4,662 prostate cancer cases and 3,114 controls.

## 2 MATERIALS AND METHODS

### 2.1 Study population

This study included 4,662 prostate cancer cases and 3,114 healthy controls from The Prostate, Lung, Colorectal, and Ovarian (PLCO) Cancer Screening Trail. Briefly, the PLCO study is a large multi‐center randomized controlled trial, the details of which have been described.^3^ Blood specimens of each participant were collected not only from the first screening visit but additional samples were also gathered during the follow‐up. The prostate cancer staging was determined in accordance with the 5^th^ edition American Joint Committee on Cancer (AJCC) staging system.

### 2.2 Gene and SNP selection from the retinol metabolism pathway

Based on the online database Kyoto Encyclopedia of Genes and Genomes (KEGG: https://www.kegg.jp/), AmiGO 2 (http://amigo.geneontology.org/amigo) and published articles,^12^, ^13^, ^14^, ^15^ a total of 31 key genes in the retinol metabolism pathway were selected (Table S1 and Figure S1).

**Figure 1:**
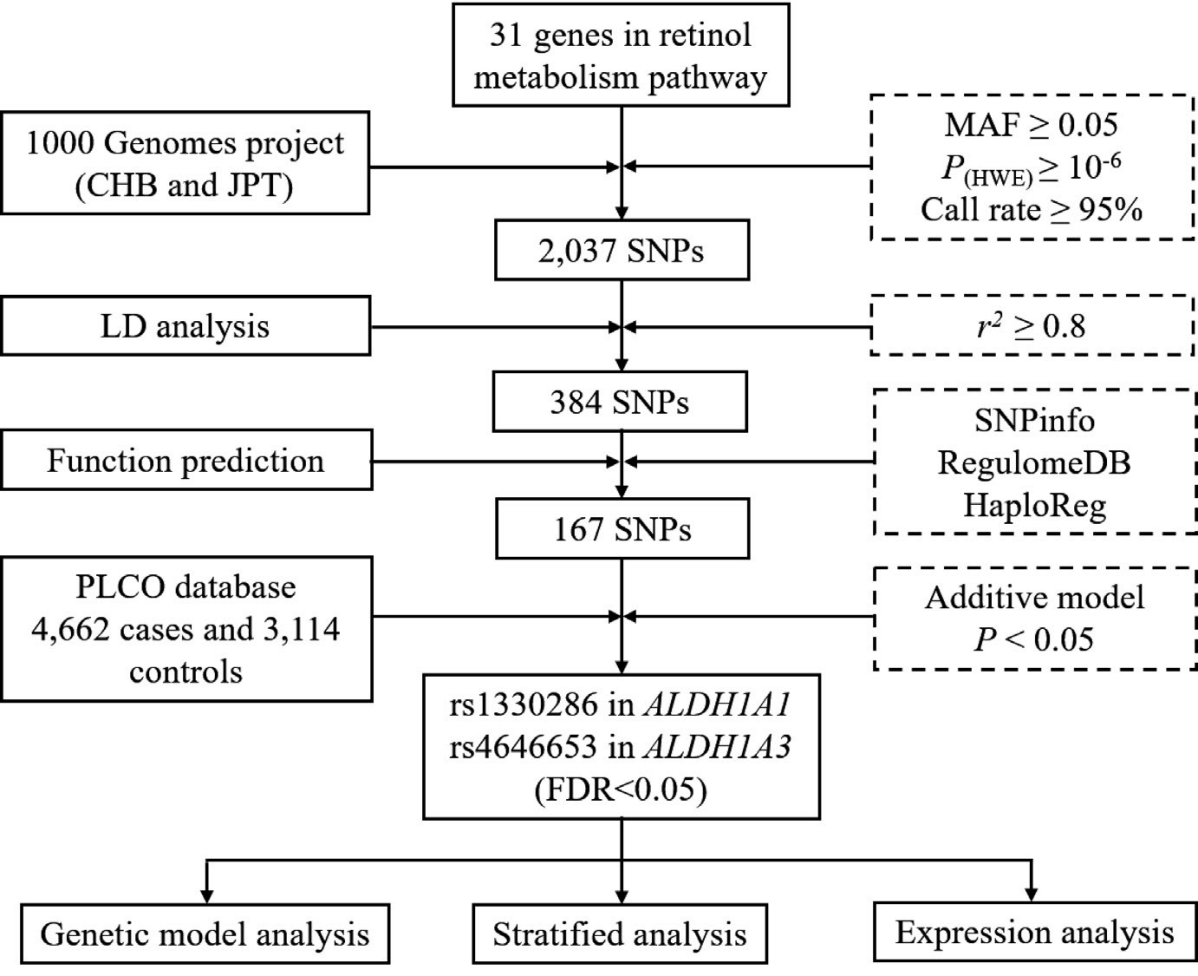
Flow chart for selecting SNPs in retinol metabolism pathway genes. *MAF* minor allele frequency, *HWE* Hardy–Weinberg equilibrium, *LD* linkage disequilibrium, *FDR* false discovery rate

The flow chart in Figure 1 shows the SNP screening process of the 31 selected genes. First, quality control was performed to identify the SNPs that reached the following requirement: minor allele frequency (MAF) ≥0.05, Hardy–Weinberg equilibrium (HWE) ≥10^−6^, and call rate ≥95%.

As a result, 2,037 genotyped SNPs were selected after quality control. Then, a linkage disequilibrium (LD) analysis was carried out using Haploview 4.2 software. We then conducted the SNP function annotation on the network tools using SNPinfo (http://snpinfo.niehs.nih.gov/snpinfo), HaploReg (http://pubs.broadinstitute.org/mammals/haploreg/haploreg.php) and RegulomeDB (http://www.regulomedb.org/). The SNPs with available predicted functions in HaploReg and a RegulomeDB score <5 were retained. In all, 167 SNPs were included for genotyping in this study.

### 2.3 SNPs genotyping

Illumina HumanHap300v1.1 and HumanHanp250 Sv1.0 were used for DNA genotyping. The genotyped data in this study were extracted from dbGap PEGASUS (phs000882) and CGEMS (phs000207). The samples and SNPs were filtered using a quality control protocol (Figure 1).

### 2.4 Statistical analysis

The Chi‐square test was applied to compare the differences between cases and controls. To evaluate the association between prostate cancer risk and genetic mutations, the adjusted odd ratios (ORs) together with their 95% confidence intervals (CIs) were calculated by an unconditional univariate and multivariate logistic regression method. The false discovery rate (FDR) was used to control the type I error due to multiple comparisons. Prostate cancer data from the GEO datasets (http://www.ncbi.nlm.nih.gov/geo/) and TCGA database (http://cancergenome.nih.gov/) were used to perform the gene expression analysis. The differences in gene expressions between prostate cancer tumor tissues and normal tissues were analyzed using a two‐sided Mann‐Whitney test. The expression quantitative trait loci (eQTL) analysis was performed using the Genotype‐Tissue Expression (GTEx) project dataset (http://www.gtexportal.org/). All statistical analyses in this study were carried out utilizing PLINK (version 1.09) and R software (version 3.2.3). A *p* value <0.05 was considered statistically significant in this study.

## 3 RESULTS

### 3.1 Characteristics of the study population

The demographic characteristics of the participants are demonstrated in Table 1. There was a significant difference in smoking status between the case group and the control group (*p* = 0.004). Of the 4,662 patients, tumor stage I/II was the largest proportion (87.23%). The percentages of Gleason score ≤6, 7, and ≥8 were 59.02%, 31.54%, and 9.44%, respectively. As for tumor aggressiveness, 2,168 cases were considered non‐aggressive and 2,040 cases were aggressive.

**Table 1:** The characteristics of study participants in the PLCO study

Characteristics	Cases (%)	Controls (%)	*p* ^a^
Number of participants	4,662	3,114	
Age^b^ (years, Mean ±SD)	68.74 ± 5.80	75.30 ± 5.36	< 0.001
Smoking status			0.004
Never	1,942 (41.66)	1,192 (38.28)	
Ever	2,355 (58.53)	1,634 (52.47)	
Current	364 (7.81)	288 (9.25)	
Missing	1	0	
Gleason score			
≤6	2,719 (59.02)		
7	1,453 (31.54)		
≥8	435 (9.44)		
Missing	55		
Stage			
I/II	4,066 (87.23)		
III/IV	595 (12.77)		
Missing	1		
Aggressiveness^c^			
Non‐aggressive	2,168 (51.52)		
Aggressive	2,040 (48.48)		
Missing	454		

Abbreviations: PLCO The Prostate, Lung, Colorectal and Ovarian Cancer Screening Trail.

^a^ *P* for Chi‐square test

^b^ Age at diagnosis for participants with prostate cancer and age at trial exit otherwise.

^c^ Aggressive: cases with a Gleason score ≥7, stage ≥III, N+, or M+

### 3.2 SNP selection and association with prostate cancer risk

We researched 167 SNPs in 24 genes in the retinol metabolism pathway for their associations with the risk of prostate cancer (Table S2). A total of six SNPs (rs1330286, rs4646653, rs4646678, rs4681028, rs4846127, and rs17016773) were discovered nominally associated with the prostate cancer risk in the additive model (*p* < 0.05). However, rs1330286 in *ALDH1A1* and rs4646653 in *ALDH1A3* were the only two SNPs that are associated with risk of prostate cancer after FDR regulation (*PFDR =0*.036 and *PFDR =0*.036, respectively; Table S3).

### 3.3 Genetic model analysis of the two SNPs

Four genetic models (additive model, dominant model, co‐dominant model, and recessive model) were employed to analyze the association between the SNPs and prostate cancer risk. For rs1330286, as shown in Table 2, the frequencies of the CC, CG, and GG genotypes of were 45.39%, 43.31%, and 11.30% in cases and 42.27%, 43.81% and 13.92% in controls. In the additive model, individuals who carry the G allele were found to have a significant decreased risk of prostate cancer compared with the individuals carrying the C allele (OR =0.88, 95% CI =0.83‐0.94, *p* = 2.45 × 10^−4^). Compared with the GC/CC genotypes, the GG genotype was significantly associated with a protective function in prostate carcinogenesis (OR = 0.79, 95% CI = 0.67‐0.93, *p* = 3.75 × 10^−3^). While for rs4646653, as shown in Table 3, an increased risk of prostate cancer was observed in individuals with the C allele (CC vs TT, OR = 1.59, 95% CI = 1.15‐2.21, *p* = 5.29 × 10^−3^). In additive model, the CC genotype has the most significant association with prostate cancer risk (OR = 1.17, 95% CI = 1.07‐1.27, *p* = 4.33 × 10^−4^). As a result, we selected the additive model for the stratified analysis of rs1330286 and rs4646653.

**Table 2:** Association between rs1330286 in *ALDH1A1* and the risk of prostate cancer

Genotypes	Cases, n (%)	Controls, n (%)	OR (95%CI)	*P*	Adjusted OR (95%CI)^a^	*P* ^a^
CC	2,114 (45.39)	1,312 (42.27)	1.00		1.00	
GC	2,017 (43.31)	1,360 (43.81)	0.92 (0.84‐1.02)	9.51 × 10^−2^	0.90 (0.80‐1.01)	6.67 × 10^−2^
GG	526 (11.30)	432 (13.92)	0.76 (0.65‐0.87)	1.48 × 10^−4^	0.75 (0.63‐0.89)	8.37 × 10^−4^
Additive model			0.88 (0.83‐0.94)	2.72 × 10^−4^	0.88 (0.83‐0.94)	2.45 × 10^−4^
Dominant model			0.88 (0.80‐0.97)	6.60 × 10^−3^	0.86 (0.78‐0.96)	7.14 × 10^−3^
Recessive model			0.79 (0.69‐0.90)	5.92 × 10^−4^	0.79 (0.67‐0.93)	3.75 × 10^−3^

Abbreviations: CI, confidence interval; OR, odds ratio.

^a^ Adjusted for age and smoking status in the logistic regression model.

**Table 3:** Association between rs4646653 in *ALDH1A3* and the risk of prostate cancer

Genotypes	Cases, n (%)	Controls, n (%)	OR (95%CI)	*P*	Adjusted OR (95%CI)^a^	*P* ^a^
TT	3,139 (67.53)	2,164 (70.53)	1.00		1.00	
CT	1,340 (28.83)	835 (27.22)	1.11 (0.99‐1.23)	5.29 × 10^−2^	1.07 (0.95‐1.21)	2.59 × 10^−1^
CC	169 (3.64)	69 (2.25)	1.69 (1.27‐2.25)	3.20 × 10^−4^	1.59 (1.15‐2.21)	5.29 × 10^−3^
Additive model			1.17 (1.07‐1.27)	4.39 × 10^−4^	1.17 (1.07‐1.27)	4.33 × 10^−4^
Dominant model		1.15 (1.04‐1.27)	5.42 × 10^−3^	1.11 (0.99‐1.25)	7.22 × 10^−2^	
Recessive model		1.64 (1.24‐2.18)	6.35 × 10^−4^	1.56 (1.13‐2.16)	7.23 × 10^−3^	

Abbreviations: CI, confidence interval; OR, odds ratio.

^a^ Adjusted for age and smoking status in the logistic regression model.

### 3.4 Stratified analysis of the two SNPs

As shown in Table 4, statistical analysis revealed that the GG genotype reduced the risk of prostate cancer only in age ≥70 years (OR = 0.87, 95% CI = 0.80‐0.94, *p* = 8.80 × 10^−4^). A subsequent stratified analysis by tumor Gleason score revealed that the GG genotype was significantly related to a decreased risk in those with a Gleason score of ≤6 and ≥8 (OR = 0.89, 95% CI = 0.82‐0.98, *p* = 1.36 × 10^−2^ and OR = 0.84, 95% CI = 0.79‐0.98, *p* = 3.15 × 10^−2^, respectively). However, similar effects were not observed in those with a Gleason score of 7 (OR = 0.91, 95% CI = 0.82‐1.01, *p* = 7.36 × 10^−2^). Furthermore, we noticed that there was a more significant association between the GG genotype and prostate cancer risk in Gleason score ≤6 than in Gleason score ≥8. When stratified by tumor stage, the association between the GG genotype and prostate cancer risk showed statistical significance in both stages I/II and stages III/IV (OR = 0.89, 95% CI = 0.82‐0.96, *p* = 3.94 × 10^−3^ and OR = 0.81, 95% CI = 0.70‐0.94, *p* = 7.07 × 10^−3^, respectively). For cancer aggressiveness, the GG genotype showed a significant decreased prostate cancer risk in both non‐aggressive cases and aggressive cases (OR = 0.89, 95% CI = 0.81‐0.97, *p* = 9.68 × 10^−3^ and OR = 0.89, 95% CI = 0.81‐0.97, *p* = 1.27 × 10^−2^, respectively).

**Table 4:** Stratified analysis for the association between rs1300286 and prostate cancer risk in the additive model

Variables	Genotypes			OR (95% CI)	*p*	Adjusted OR (95% CI)^a^	*p* ^a^
	GG, n (%)	CG, n (%)	CC, n (%)				
Controls	432 (13.92)	1,360 (43.81)	1,312 (42.27)	1.00	1.00	1.00	
Cases	526 (11.29)	2,017 (43.31)	2,114 (45.40)	0.88 (0.83‐0.94)	2.72 × 10^−4^	0.88 (0.83‐0.94)	2.45 × 10^−4^
Age							
<70	293 (11.42)	1,123 (43.76)	1,150 (44.82)	0.91 (0.80‐1.05)	1.92 × 10^−1^	0.87 (0.76‐1.00)	5.82 × 10^−2^
≥70	233 (11.14)	894 (42.76)	964 (46.10)	0.86 (0.79‐0.94)	7.65 × 10^−4^	0.87 (0.80‐0.94)	8.80 × 10^−4^
Gleason score							
≤6	306 (11.27)	1,166 (42.93)	1,244 (45.80)	0.88 (0.81‐0.94)	5.88 × 10^−4^	0.89 (0.82‐0.98)	1.36 × 10^−2^
7	166 (11.43)	648 (44.63)	638 (43.94)	0.92 (0.84‐1.00)	5.69 × 10^−2^	0.91 (0.82‐1.01)	7.36 × 10^−2^
≥8	47 (10.83)	181 (41.71)	206 (47.46)	0.84 (0.72‐0.97)	1.96 × 10^−2^	0.84 (0.79‐0.98)	3.15 × 10^−2^
Stage							
I/II	459 (11.30)	1,778 (43.78)	1,824 (44.92)	0.89 (0.83‐0.96)	1.20 × 10^−3^	0.89 (0.82‐0.96)	3.94 × 10^−3^
III/IV	67 (11.26)	238 (40.00)	290 (48.74)	0.82 (0.72‐0.94)	3.26 × 10^−3^	0.81 (0.70‐0.94)	7.07 × 10^−3^
Aggressiveness							
Non‐aggressive	240 (11.35)	931 (44.02)	944 (44.63)	0.88 (0.81‐0.94)	7.40 × 10^−4^	0.89 (0.81‐0.97)	9.68 × 10^−3^
Aggressive	231 (11.38)	892 (43.75)	915 (44.87)	0.90 (0.82‐0.97)	7.66 × 10^−3^	0.89 (0.81‐0.97)	1.27 × 10^−2^

Abbreviations: CI, confidence interval; OR, odds ratio.

^a^ Adjusted for age and smoking status in the logistic regression model.

In stratification analysis of rs4646653, which was also stratified by age, Gleason score, stage and aggressiveness, a significant increased prostate cancer risk effect was found in age ≥70, Gleason score in 7, stages I/II and aggressive cases (OR = 1.16, 95% CI = 1.04‐1.29, *p* = 6.65 × 10^−3^; OR = 1.22, 95% CI = 1.07‐1.39, *p* = 3.91 × 10^−3^; OR = 1.12, 95% CI = 1.01‐1.24, *p* = 3.08 × 10^−2^, and OR = 1.18, 95% CI = 1.05‐1.33, *p* = 6.02 × 10^−3^, respectively, Table 5).

**Table 5:** Stratified analysis for the association between rs4646653 and prostate cancer risk in the additive model

Variables	Genotypes			OR (95% CI)	*p*	Adjusted OR (95% CI)^a^	*p* ^a^
	CC, n (%)	CT, n (%)	TT, n (%)				
Controls	69 (2.25)	835 (27.22)	2,164 (70.53)	1.00		1.00	
Cases	169 (3.64)	1,340 (28.83)	3,139 (67.53)	1.17 (1.07‐1.27)	4.39 × 10^−4^	1.17 (1.07‐1.27)	4.33 × 10^−4^
Age							
<70	86 (3.36)	750 (29.32)	1722 (67.32)	0.94 (0.79‐1.11)	4.38 × 10^−1^	0.94 (0.79‐1.12)	4.79 × 10^−1^
≥70	83 (3.97)	590 (28.23)	1417 (67.80)	1.23 (1.10‐1.37)	2.20 × 10^−4^	1.16 (1.04‐1.29)	6.65 × 10^−3^
Gleason score							
≤6	95 (3.51)	768 (28.35)	1846 (68.14)	1.14 (1.03‐1.26)	8.87 × 10^−3^	1.06 (0.94‐1.19)	3.65 × 10^−1^
7	58 (4.00)	435 (30.00)	957 (66.00)	1.25 (1.11‐1.40)	1.98 × 10^−4^	1.22 (1.07‐1.39)	3.91 × 10^−3^
≥8	16 (3.69)	120 (27.65)	298 (68.66)	1.13 (0.93‐1.36)	2.12 × 10^−1^	1.06 (0.87‐1.29)	5.93 × 10^−1^
Stage							
I/II	142 (3.50)	1169 (28.83)	2744 (67.67)	1.16 (1.06‐1.27)	1.25 × 10^−3^	1.12 (1.01‐1.24)	3.08 × 10^−2^
III/IV	27 (4.56)	171 (28.89)	394 (66.55)	1.25 (1.06‐1.46)	7.39 × 10^−3^	1.12 (0.93‐1.36)	2.33 × 10^−1^
Aggressiveness							
Non‐aggressive	77 (3.57)	621 (28.73)	1463 (67.70)	1.16 (1.05‐1.29)	5.15 × 10^−3^	1.05 (0.93‐1.18)	4.31 × 10^−1^
Aggressive	79 (3.88)	595 (29.26)	1360 (66.86)	1.21 (1.09‐1.34)	4.83 × 10^−4^	1.18 (1.05‐1.33)	6.02 × 10^−3^

Abbreviations: CI, confidence interval; OR, odds ratio.

^a^ Adjusted for age and smoking status in the logistic regression model.

### 3.5 Expression quantitative trait loci analysis

Furthermore, we practiced an eQTL analysis to evaluate the effects of rs1330286 in *ALDH1A1* and rs4646653 in *ALDH1A3* from the GTEx dataset. As illustrated in Figure S2, rs1330286 was significantly related to the expression of *ALDH1A1* in 132 prostate tissue samples (*p* = 2.90 × 10^−3^). Data of rs4646653 were not available in this dataset.

### 3.6 Expression levels of *ALDH1A1* and *ALDH1A3* in prostate cancer and normal tissues

**Figure 2:**
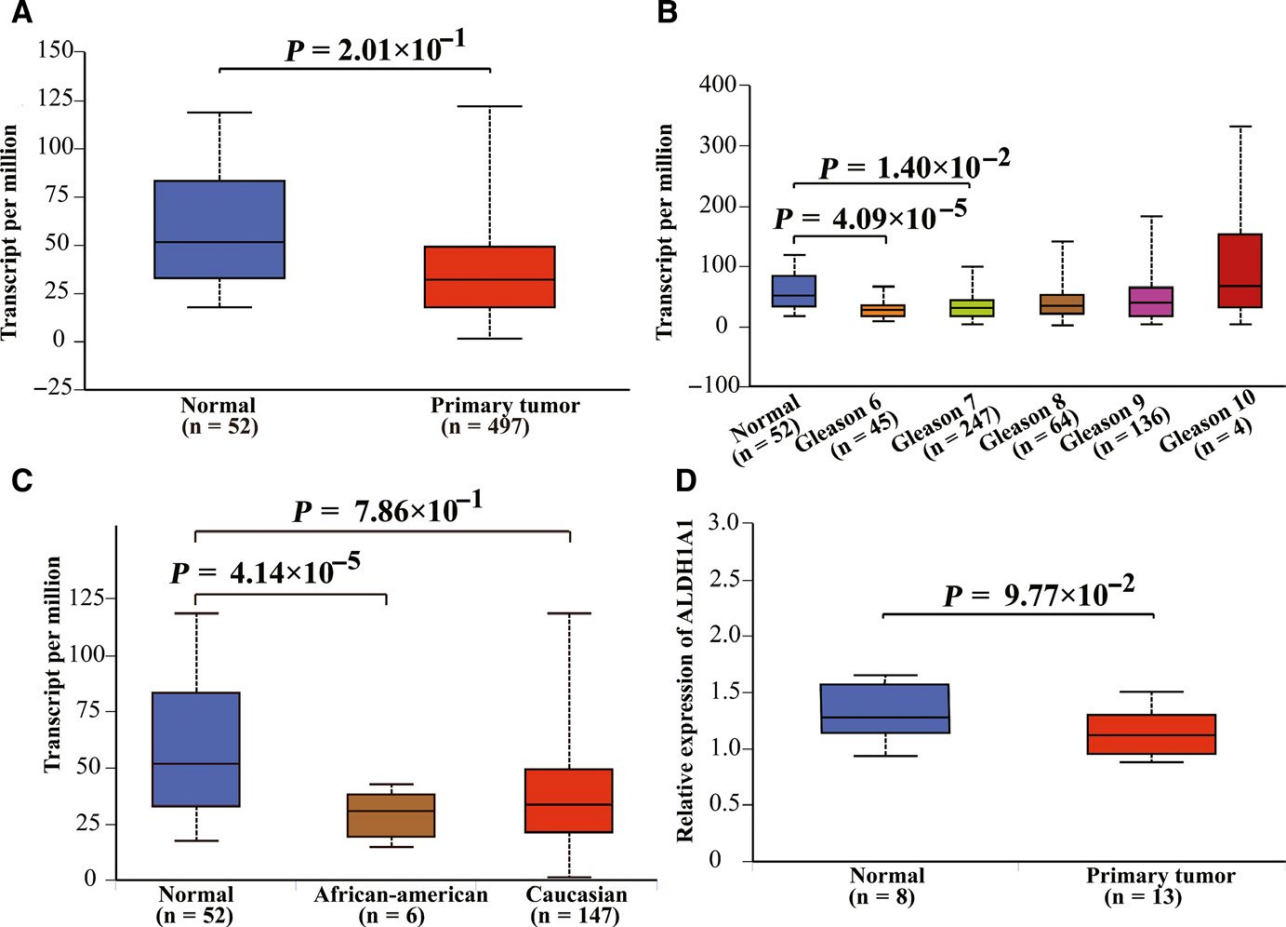
There is no difference between the expression level of *ALDH1A1* in prostate cancer tumors and normal tissues. The relative expression levels of *ALDH1A1* in TCGA database (A, B, and C) and GEO database (GSE55945) (D)

**Figure 3:**
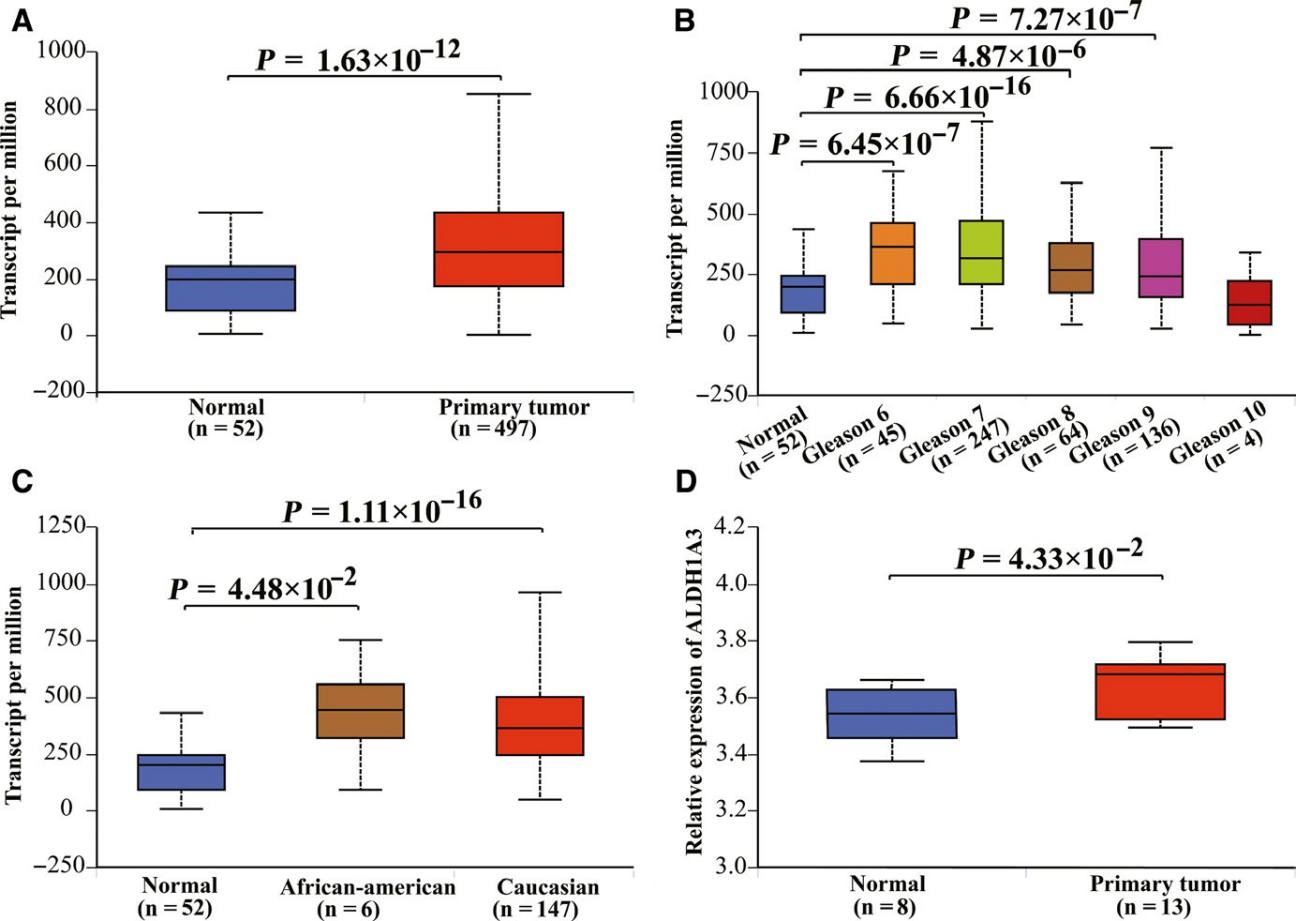
The expression level of *ALDH1A3* in prostate cancer tumors is significantly higher than in normal tissues. The relative expression levels of *ALDH1A3* in TCGA database (A, B, and C) and GEO database (GSE55945) (D)

Data from TCGA datasets and GEO datasets were used for gene expression analysis. There was no significant difference in mRNA transcription levels of *ALDH1A1* between prostate tumor tissues and normal tissues (*p* = 2.01 × 10^−1^ in TCGA datasets and *p* = 9.77 × 10^−2^ in GSE55945 datasets, respectively; Figure 2). When stratified by ethnicity and Gleason score, the *ALDH1A1* transcription level in African‐American cases was significantly lower than that in normal tissues (*p* = 4.14 × 10^−5^), so was the tumor tissues in Gleason 6 and 7 (*p* = 4.09 × 10^−5^ and *p* = 1.40 × 10^−2^). When it comes to *ALDH1A3*, as shown in Figure 3, the mRNA transcription level of *ALDH1A3* was significantly higher in prostate cancer tissues than that in normal tissues in both TCGA datasets and GEO datasets (*p* = 1.63 × 10^−12^ and *p* = 4.33 × 10^−2^, respectively). Moreover, when stratified by race, both Caucasian and African‐American patients have higher mRNA transcription level of *ALDH1A3* compared to normal tissues. Meanwhile, the same outcomes are also found in patients with a Gleason score of 6 to 9.

## 4 DISCUSSION

Retinol, the biologically active form of vitamin A, has an effect in plenty of biological processes including normal growth and development,^16^ tissue homeostasis maintenance,^17^ and protection from diseases.^18^ Retinol plays its effect of gene transcription regulation via RARs and RXRs, which act as the nuclear DNA‐binding receptors.^19^ It has been demonstrated that retinol can inhibit the development of different types of tumors,^20^ such as breast, skin, lung, and prostate cancers. A published study suggested that high serum retinol was associated with increased prostate cancer risk.^21^ Moreover, high concentrations of serum retinol may have an adverse effect on prostate through the insulin‐like growth factor I receptor^22^ or by antagonizing vitamin D.^23^ Genetic mutation of genes involved in the retinol metabolism pathway has also aroused a lot of interest in recent years.

In this present study, we investigated whether SNPs of genes in the retinol metabolism pathway are associated with prostate cancer risk utilizing available data from the PLCO trail. After adjusting for age and smoking status, we identified that the rs1330286 G allele in *ALDH1A1* was associated with a decreased risk of prostate cancer, while the rs4646653 C allele in *ALDH1A3* was strongly related to an increased risk of prostate cancer. Gene expression analysis revealed that the expression of *ALDH1A3* was significantly higher in the prostate cancer tumor tissues than that in the normal ones. Moreover, rs1330286 was found a significant eQTL of *ALDH1A1*.

*ALDH1A1*, known as a member of the aldehyde dehydrogenase family, plays a role in the production of retinoic acid in cells. Aldehyde dehydrogenase (*ALDH*) is a superfamily of enzymes consisting of 19 isoforms, which are involved in the catabolism of aldehydes agents, stem cell protection, and differentiation.^24^ Previous studies have shown that aldehyde dehydrogenase has a potential function of antioxidant, thus maintaining stemness in cells. *ALDH1A1* is a main member of the *ALDH* superfamily that catabolizes the oxidation of intracellular aldehydes, oxidizing retinol to retinoic acid (RA) through an alcohol intermediary, and it plays an important role in stem cell differentiation and protection. The expression level of *ALDH1A1* in prostate cancer was significantly different from that in benign prostate hyperplasia samples.^25^ In our study, one SNP in the *ALDH1A1* intron region, which has an annotated function of changing motifs, is associated with prostate cancer risk. However, we failed to find a significant difference in *ALDH1A1* expression between the prostate cancer tumor tissues and the normal tissues. This result may be explained by the limitation of sample size, indicating that more tissue samples are needed to confirm the effects of *ALDH1A1*.

*ALDH1A3*, another member of the ALDH superfamily, is found highly expressed in many different cancers, such as ovarian cancer and pancreatic cancer.^26^ In colorectal cancer, *ALDH1A3* upregulation is associated with acquired chemoresistance and metastatic dissemination.^27^ A study reported that *ALDH1A3 *has a high expression in prostate cancer and is associated with progression‐free survival after prostatectomy.^28^ In this study, we discovered that rs4646653 in *ALDH1A3* is related to an increased risk of prostate cancer. Moreover, compared to the expression level in normal prostate tissues, the expression level of *ALDH1A3* is significantly higher in prostate cancer tumor tissues. Although there are some important discoveries revealed by this study, there are some limitations as well. First, apart from SNPs, other types of genetic variants such as InDel, CNV, and rearrangement could also contribute to the carcinogenesis of prostate cancer, but they were not investigated in this study. Further researches could touch this area. Second, we failed to clarify whether rs4646653 was related to the expression of *ALDH1A3*, for the eQTL data was not available on GTEx website. Further biological researches are needed to clarify the function of retinol‐related genes in the carcinogenesis of prostate cancer.

In conclusion, our study demonstrated that the genetic variants in *ALDH1A1* and *ALDH1A3* may play an important role in the tumorigenesis of prostate cancer. These results may offer more clarified evidence of the association of retinol metabolism pathway genes and prostate cancer carcinogenesis and development.

## CONFLICT OF INTEREST

None declared.

## AUTHOR CONTRIBUTIONS

Gong Cheng and Lixin Hua designed the study. Meilin Wang, Shuai Ben, and Yifei Cheng contributed to the data collection. Junyi Xin and Shuwei Li performed the data analysis. Dongliang Cao and Yixuan Meng interpreted the data and wrote the manuscript. All authors read and approved the final manuscript.

## Supporting information

Supplementary Material

## Data Availability

The data are available on request.
