# Supplementary material for: Association study between genetic variants in retinol metabolism pathway genes and prostate cancer risk
Source: Cancer Med. 2020 Oct 17;9(24):9462–70. doi: 10.1002/cam4.3538 (PMC7774741; doi:10.1002/cam4.3538)
Supplement: Supplementary file 1 — Supplementary Material [file CAM4-9-9462-s001.docx]

**Supplementary Table 1.** **List of retinol metabolism pathway genes**

| Gene | Chromosome | Location | Start | End | Description |
| --- | --- | --- | --- | --- | --- |
| *DHRS3* | 1 | NC_000001.10 | 12627939 | 12677820 | dehydrogenase/reductase 3 |
| *RPE65* | 1 | NC_000001.10 | 68894507 | 68915642 | retinoid isomerohydrolase |
| *RXRG* | 1 | NC_000001.10 | 165370159 | 165414592 | retinoid X receptor gamma |
| *AOX1* | 2 | NC_000002.11 | 201450731 | 201536218 | aldehyde oxidase 1 |
| *CYP26B1* | 2 | NC_000002.11 | 72356367 | 72374991 | cytochrome P450 family 26 subfamily B member 1 |
| *DHRS9* | 2 | NC_000002.11 | 169921299 | 169952677 | dehydrogenase/reductase 9 |
| *RDH14* | 2 | NC_000002.11 | 18735989 | 18741959 | retinol dehydrogenase 14 |
| *RARB* | 3 | NC_000003.11 | 25215638 | 25639423 | retinoic acid receptor beta |
| *ADH4* | 4 | NC_000004.11 | 100044832 | 100065449 | alcohol dehydrogenase 4 |
| *ADH7* | 4 | NC_000004.11 | 100333418 | 100356667 | alcohol dehydrogenase 7 |
| *LRAT* | 4 | NC_000004.11 | 155665163 | 155674270 | lecithin retinol acyltransferase |
| *RXRB* | 6 | NC_000006.11 | 33161362 | 33168630 | retinoid X receptor beta |
| *DGAT1* | 8 | NC_000008.10 | 145538246 | 145550582 | diacylglycerol O-acyltransferase 1 |
| *RDH10* | 8 | NC_000008.10 | 74206837 | 74237520 | retinol dehydrogenase 10 |
| *SDR16C5* | 8 | NC_000008.10 | 57212570 | 57233335 | short chain dehydrogenase/reductase family 16C member 5 |
| *ALDH1A1* | 9 | NC_000009.11 | 75515578 | 75653633 | aldehyde dehydrogenase 1 family member A1 |
| *RXRA* | 9 | NC_000009.11 | 137218316 | 137332431 | retinoid X receptor alpha |
| *CYP26A1* | 10 | NC_000010.10 | 94833232 | 94837641 | cytochrome P450 family 26 subfamily A member 1 |
| *CYP26C1* | 10 | NC_000010.10 | 94820565 | 94829304 | cytochrome P450 family 26 subfamily C member 1 |
| *RBP4* | 10 | NC_000010.10 | 95351593 | 95361101 | retinol binding protein 4 |
| *RARG* | 12 | NC_000012.11 | 53604350 | 53626040 | retinoic acid receptor gamma |
| *RDH16* | 12 | NC_000012.11 | 57345215 | 57353158 | retinol dehydrogenase 16 |
| *RDH5* | 12 | NC_000012.11 | 56114151 | 56118526 | retinol dehydrogenase 5 |
| *RDH11* | 14 | NC_000014.8 | 68143517 | 68162510 | retinol dehydrogenase 11 |
| *RDH12* | 14 | NC_000014.8 | 68168603 | 68201168 | retinol dehydrogenase 12 |
| *ALDH1A2* | 15 | NC_000015.9 | 58245622 | 58358121 | aldehyde dehydrogenase 1 family member A2 |
| *ALDH1A3* | 15 | NC_000015.9 | 101419897 | 101456831 | aldehyde dehydrogenase 1 family member A3 |
| *BCO1* | 16 | NC_000016.9 | 81272176 | 81324747 | beta-carotene oxygenase 1 |
| *RARA* | 17 | NC_000017.10 | 38465423 | 38513895 | retinoic acid receptor alpha |
| *RDH13* | 19 | NC_000019.9 | 55555684 | 55580914 | retinol dehydrogenase 13 |
| *RDH8* | 19 | NC_000019.9 | 10123925 | 10132954 | retinol dehydrogenase 8 |

**Supplementary Table 2. Function annotation of 167 SNPs**

| SNPs | Gene | Location | SNPinfo | Regulome DB score | HaploReg v4.1 | | | | |
| --- | --- | --- | --- | --- | --- | --- | --- | --- | --- |
|  |  |  |  |  | Promoter histone marks | Enhancer histone marks | DNase | Motifs changed | eQTL hits |
| rs4075304 | *DHRS3* | -- | -- | 4 | -- | LNG, BLD | -- | LUN-1 | -- |
| rs4072868 | *DHRS3* | intronic | -- | 2b | -- | LNG | -- | 4 altered motifs | 1 hit |
| rs3128451 | *DHRS3* | intronic | -- | 4 | -- | 10 tissues | SKIN,SKIN | -- | 1 hit |
| rs3128449 | *DHRS3* | intronic | -- | 4 | -- | 10 tissues | ADRL | SP1 | 1 hit |
| rs4846127 | *DHRS3* | intronic | -- | 3a | CRVX | 21 tissues | -- | Bbx,STAT | -- |
| rs4394668 | *DHRS3* | intronic | -- | 1f | -- | 8 tissues | PLCNT,BLD | Cart1 | -- |
| rs5022243 | *DHRS3* | intronic | -- | 4 | -- | 7 tissues | BLD,BLD | Pbx-1 | 1 hit |
| rs11121951 | *DHRS3* | intronic | -- | 1f | 22 tissues | 5 tissues | 38 tissues | LXR,Znf143 | 1 hit |
| rs3125899 | *RPE65* | intronic | -- | 5 | -- | BLD | -- | 8 altered motifs | 1 hit |
| rs17130694 | *RPE65* | intronic | -- | 4 | -- | 6 tissues | 14 tissues | STAT,TATA | -- |
| rs3790472 | *RPE65* | intronic | -- | 5 | -- | -- | -- | Pou3f2,Sox,p300 | -- |
| rs283700 | *RXRG* | intronic | -- | 2b | BLD | 7 tissues | 7 tissues | AP-1,CACD,TEF-1 | -- |
| rs10800096 | *RXRG* | intronic | -- | 7 | -- | -- | -- | NRSF,PRDM1 | -- |
| rs283694 | *RXRG* | intronic | -- | 4 | -- | FAT, SKIN, MUS | -- | NRSF,Zic | -- |
| rs2651860 | *RXRG* | intronic | -- | 5 | -- | HRT | -- | MAZ | -- |
| rs12069160 | *RXRG* | intronic | -- | 5 | -- | -- | -- | Ik-2 | -- |
| rs10489745 | *RXRG* | intronic | -- | 5 | -- | -- | -- | Hic1,Pax-5 | -- |
| rs188196 | *RXRG* | intronic | -- | 5 | -- | -- | -- | 4 altered motifs | 1 hit |
| rs6676763 | *RXRG* | intronic | -- | 5 | -- | -- | -- | CTCF,Myc,Pax-4 | -- |
| rs283691 | *RXRG* | intronic | -- | 7 | -- | -- | -- | 4 altered motifs | -- |
| rs10918180 | *RXRG* | intronic | -- | -- | -- | -- | -- | ATF3,HES1,Rad21 | -- |
| rs74118603 | *RXRG* | intronic | -- | 7 | -- | ESDR, MUS | -- | 8 altered motifs | -- |
| rs1831002 | *RXRG* | intronic | -- | 7 | -- | LNG, MUS | -- | 4 altered motifs | -- |
| rs752739 | *RXRG* | intronic | -- | 4 | 7 tissues | 10 tissues | 4 tissues | 5 altered motifs | -- |
| rs1467664 | *RXRG* | -- | TFBS | 4 | 5 tissues | 9 tissues | HRT,KID,LNG | 8 altered motifs | -- |
| rs16985278 | *RDH14* | -- | -- | 4 | -- | 14 tissues | 4 tissues | -- | -- |
| rs13403096 | *RDH14* | -- | -- | 5 | -- | SKIN, HRT | -- | Mef2,STAT,p300 | -- |
| rs707718 | *CYP26B1* | 3'-UTR | miRNA | 4 | -- | ESDR, ESC, THYM | -- | HMG-IY,Pou2f2,Zfp105 | 1 hit |
| rs6753502 | *DHRS9* | intronic | -- | 7 | BLD | 7 tissues | SKIN | Nkx2,TCF11::MafG | 4 hits |
| rs17532798 | *AOX1* | intronic | -- | 4 | LIV | 4 tissues | -- | Irf | -- |
| rs1527945 | *AOX1* | intronic | -- | 4 | LIV | 6 tissues | -- | 7 altered motifs | 2 hits |
| rs12993144 | *AOX1* | intronic | -- | 5 | -- | SKIN, ADRL, CRVX | -- | 18 altered motifs | 1 hit |
| rs13406379 | *AOX1* | intronic | -- | 2b | -- | 11 tissues | MUS | 12 altered motifs | 2 hits |
| rs16833889 | *AOX1* | intronic | -- | 4 | SKIN, LNG | 15 tissues | SKIN,BRN | -- | 2 hits |
| rs59699466 | *AOX1* | intronic | -- | 7 | -- | FAT, SKIN | -- | NF-kappaB,SIX5,ZBTB33 | 1 hit |
| rs6732225 | *AOX1* | intronic | -- | 4 | -- | 9 tissues | 15 tissues | STAT,WT1 | 1 hit |
| rs6720189 | *AOX1* | intronic | -- | 1f | -- | 10 tissues | -- | AP-3 | 1 hit |
| rs1986415 | *AOX1* | intronic | -- | 5 | -- | 6 tissues | -- | Pax-6 | -- |
| rs2254576 | *AOX1* | intronic | -- | 3a | -- | ESDR | -- | GR,Hltf,TATA | -- |
| rs2465661 | *AOX1* | intronic | -- | 5 | BLD | 4 tissues | -- | RXRA | 2 hits |
| rs7562507 | *AOX1* | intronic | -- | 3a | -- | ESDR, ADRL, MUS | ADRL,MUS | CHOP::CEBPalpha | -- |
| rs6435060 | *AOX1* | intronic | -- | 4 | -- | ADRL | -- | -- | -- |
| rs4674311 | *AOX1* | intronic | -- | 4 | -- | BLD, CRVX, BRN | -- | -- | -- |
| rs2715905 | *AOX1* | intronic | -- | 4 | -- | BLD | -- | Irf,SIX5 | -- |
| rs2540066 | *AOX1* | intronic | -- | 4 | -- | BLD, THYM | THYM | Ik-1,Znf143 | -- |
| rs9825855 | *RARB* | -- | -- | 7 | -- | -- | -- | 7 altered motifs | -- |
| rs1483846 | *RARB* | -- | -- | 7 | -- | -- | -- | 5 altered motifs | -- |
| rs1436252 | *RARB* | -- | -- | 2b | -- | 9 tissues | 13 tissues | Mtf1,Smad4 | -- |
| rs73154973 | *RARB* | -- | -- | 3a | -- | ESDR, BLD | -- | AP-1,Egr-1,Nanog | -- |
| rs977226 | *RARB* | -- | -- | 7 | -- | -- | -- | Evi-1,Foxa | -- |
| rs2164478 | *RARB* | -- | -- | 3a | -- | ESDR, ESC, IPSC | -- | 8 altered motifs | -- |
| rs1483836 | *RARB* | -- | -- | 5 | -- | ESDR | -- | CEBPB,SRF | -- |
| rs74347859 | *RARB* | -- | -- | 5 | -- | ESDR | -- | Maf,RFX5 | -- |
| rs1483830 | *RARB* | -- | -- | 5 | -- | -- | -- | AP-1,Maf,VDR | -- |
| rs1075410 | *RARB* | -- | -- | 5 | -- | -- | -- | 8 altered motifs | -- |
| rs1483843 | *RARB* | -- | -- | 7 | -- | -- | -- | Sox | -- |
| rs13088742 | *RARB* | -- | -- | 4 | -- | ESDR, BLD, GI | -- | Myb,YY1 | -- |
| rs2116701 | *RARB* | -- | -- | 5 | -- | GI | -- | Bcl6b,STAT | -- |
| rs59648542 | *RARB* | -- | -- | 7 | -- | -- | -- | SZF1-1,Spz1 | -- |
| rs6783726 | *RARB* | -- | -- | 5 | -- | MUS | -- | 5 altered motifs | -- |
| rs4681060 | *RARB* | -- | -- | 7 | -- | GI | -- | Ets | -- |
| rs6550974 | *RARB* | -- | -- | 5 | -- | -- | -- | 6 altered motifs | -- |
| rs9865116 | *RARB* | -- | -- | 2b | IPSC | 5 tissues | 6 tissues | 6 altered motifs | -- |
| rs6778608 | *RARB* | -- | -- | 4 | IPSC | 5 tissues | 6 tissues | Foxa,STAT | -- |
| rs755661 | *RARB* | intronic | -- | 2b | 18 tissues | 4 tissues | 12 tissues | 10 altered motifs | -- |
| rs4681025 | *RARB* | intronic | -- | 4 | LNG, VAS, BLD | 7 tissues | HRT,LNG,VAS | RREB-1 | -- |
| rs9284856 | *RARB* | intronic | -- | 5 | LNG | 12 tissues | SKIN | 9 altered motifs | 1 hit |
| rs12631063 | *RARB* | intronic | -- | 4 | -- | LNG | -- | -- | -- |
| rs13314219 | *RARB* | intronic | -- | 4 | 8 tissues | 16 tissues | 12 tissues | 5 altered motifs | -- |
| rs6778350 | *RARB* | intronic | -- | 2a | -- | 7 tissues | -- | GR,LUN-1 | -- |
| rs1529672 | *RARB* | intronic | -- | 4 | -- | 9 tissues | ESDR,PLCNT | RXRA,p300 | -- |
| rs112050806 | *RARB* | intronic | -- | 5 | -- | HRT, PLCNT | -- | HEY1 | -- |
| rs1881706 | *RARB* | intronic | -- | 4 | -- | 12 tissues | 13 tissues | Nanog | -- |
| rs1153588 | *RARB* | intronic | -- | 3a | BRN | 13 tissues | 12 tissues | 9 altered motifs | -- |
| rs17526019 | *RARB* | intronic | -- | 5 | -- | 4 tissues | -- | -- | -- |
| rs1286665 | *RARB* | intronic | -- | 4 | -- | 4 tissues | GI,GI | -- | -- |
| rs1881703 | *RARB* | intronic | -- | 5 | -- | 6 tissues | 4 tissues | RXRA | -- |
| rs17016738 | *RARB* | intronic | -- | 5 | STRM | 5 tissues | BRN,LNG | AP-2 | -- |
| rs17016773 | *RARB* | intronic | -- | 5 | -- | -- | -- | CEBPB | -- |
| rs77132200 | *RARB* | intronic | -- | -- | -- | HRT | -- | SP1 | -- |
| rs1286733 | *RARB* | intronic | -- | 4 | -- | -- | -- | CTCF,Smad3 | -- |
| rs1286734 | *RARB* | intronic | -- | 4 | -- | BONE | IPSC | Gfi1,SRF | -- |
| rs1286738 | *RARB* | intronic | -- | 4 | MUS, BRN, BONE | 14 tissues | 6 tissues | NRSF | -- |
| rs4681028 | *RARB* | intronic | -- | 2b | -- | 5 tissues | 8 tissues | 6 altered motifs | 1 hit |
| rs7621140 | *RARB* | intronic | -- | 4 | LNG | 4 tissues | SKIN,HRT | BCL,NRSF,PU.1 | 51 hits |
| rs1058378 | *RARB* | 3'-UTR | Splicing, miRNA | -- | -- | -- | GI | HNF1 | 66 hits |
| rs2032348 | *ADH4* | intronic | -- | 7 | LIV, GI | BRN | -- | Pou1f1,Pou2f2 | 11 hits |
| rs1800761 | *ADH4* | intronic | -- | 4 | FAT, LIV, GI | 9 tissues | 7 tissues | -- | 17 hits |
| rs4148884 | *ADH4* | intronic | TFBS | 5 | -- | 4 tissues | -- | -- | 3 hits |
| rs2851012 | *ADH7* | -- | -- | 5 | -- | IPSC, SKIN | -- | 5 altered motifs | -- |
| rs2584463 | *ADH7* | -- | -- | 7 | -- | BRST | -- | 4 altered motifs | -- |
| rs284786 | *ADH7* | 3'-UTR | -- | 7 | -- | FAT, LIV | -- | Mef2 | -- |
| rs4147551 | *ADH7* | intronic | -- | 4 | -- | FAT, LIV, GI | GI,BRST | -- | -- |
| rs1827567 | *ADH7* | intronic | -- | 5 | LIV | FAT, GI | -- | RREB-1 | -- |
| rs1154456 | *ADH7* | intronic | -- | 7 | -- | -- | -- | E2F,HDAC2,Nanog | -- |
| rs1154458 | *ADH7* | intronic | -- | 7 | -- | -- | -- | HNF4,Sin3Ak-20 | -- |
| rs1154460 | *ADH7* | intronic | -- | 7 | -- | -- | -- | Nanog,Pou2f2,Pou3f3 | -- |
| rs201824 | *LRAT* | intronic | -- | 7 | 4 tissues | -- | -- | Pax-5,ZEB1 | -- |
| rs78274322 | *LRAT* | 3'-UTR | -- | 7 | -- | -- | -- | Pou2f2 | -- |
| rs1546877 | *RXRB* | -- | TFBS | 4 | 13 tissues | 11 tissues | 9 tissues | -- | 11 hits |
| rs2076310 | *RXRB* | intronic | TFBS | 1f | 5 tissues | 21 tissues | LIV | 4 altered motifs | 32 hits |
| rs13279861 | *SDR16C5* | -- | -- | 4 | -- | -- | -- | E4BP4 | -- |
| rs4419802 | *SDR16C5* | intronic | -- | 1f | -- | 5 tissues | 4 tissues | -- | -- |
| rs7819550 | *RPL7* | -- | TFBS | 2b | 24 tissues | -- | 52 tissues | 5 altered motifs | 1 hit |
| rs35680921 | *RDH10* | intronic | TFBS | 4 | 20 tissues | 6 tissues | 20 tissues | FAC1,Pax-4,SREBP | 2 hits |
| rs4737370 | *RDH10* | intronic | -- | 4 | -- | FAT, LIV, BLD | 15 tissues | -- | -- |
| rs2925455 | *RDH10* | intronic | -- | 5 | -- | 9 tissues | -- | Arid5a,Cdx2,Hoxb8 | -- |
| rs4738319 | *RDH10* | intronic | -- | 5 | -- | 4 tissues | GI | Foxp1,TATA | 1 hit |
| rs17215061 | *RDH10* | intronic | -- | 7 | -- | BLD, SKIN | OVRY | INSM1,Rad21 | 9 hits |
| rs348482 | *ALDH1A1* | -- | -- | 3a | -- | BRST, GI, LNG | LNG | 6 altered motifs | 7 hits |
| rs168351 | *ALDH1A1* | intronic | -- | 3a | -- | LNG | LNG | ZEB1 | 4 hits |
| rs4646547 | *ALDH1A1* | intronic | -- | #-- | -- | LNG | LNG | EBF | -- |
| rs348461 | *ALDH1A1* | intronic | -- | 3a | -- | SKIN, LIV, PANC | LNG,CRVX | 16 altered motifs | 1 hit |
| rs13959 | *ALDH1A1* | synonymous | Splicing | 4 | -- | SKIN, LIV, PANC | 8 tissues | -- | -- |
| rs1330286 | *ALDH1A1* | intronic | -- | 5 | LIV | 5 tissues | PANC,LNG,MUS | Mrg,ZEB1 | 2 hits |
| rs10869206 | *ALDH1A1* | -- | -- | 3a | -- | -- | 13 tissues | Myf,Nkx2,Nkx3 | 2 hits |
| rs62560451 | *ALDH1A1* | -- | -- | 3a | LNG | 8 tissues | 12 tissues | Hsf,STAT,Zfp187 | 2 hits |
| rs7860374 | *ALDH1A1* | -- | -- | 5 | -- | -- | -- | HNF1,TEF | 1 hit |
| rs2310308 | *ALDH1A1* | -- | -- | 7 | -- | ESDR, PANC | PANC | -- | -- |
| rs11102986 | *RXRA* | intronic | -- | 5 | BLD | 17 tissues | BLD,BLD,BLD | EWSR1-FLI1,Klf7 | -- |
| rs12115573 | *RXRA* | intronic | -- | 2c | 7 tissues | 17 tissues | 18 tissues | NF-kappaB,RFX5 | -- |
| rs12004589 | *RXRA* | intronic | -- | 4 | BLD | 12 tissues | -- | SRF,Znf143 | -- |
| rs3118529 | *RXRA* | intronic | -- | 3a | MUS | 22 tissues | PLCNT,MUS,BRST | 4 altered motifs | -- |
| rs877954 | *RXRA* | intronic | -- | 4 | 4 tissues | 21 tissues | 14 tissues | Nkx3 | -- |
| rs1538648 | *CYP26C1* | -- | TFBS | 2b | ESC, ESDR, IPSC | 14 tissues | 7 tissues | GR,Mef2 | -- |
| rs4919592 | *CYP26C1* | intronic | -- | 4 | ESC, ESDR, IPSC | FAT, BLD, SKIN | SKIN | -- | -- |
| rs4411227 | *CYP26A1* | -- | TFBS | 4 | 10 tissues | 11 tissues | 4 tissues | 6 altered motifs | 2 hits |
| rs11187541 | *RBP4* | -- | -- | 2b | -- | 7 tissues | PLCNT | 13 altered motifs | 1 hit |
| rs7094671 | *RBP4* | intronic | -- | 4 | -- | 7 tissues | ESDR,BRST | AP-2 | 1 hit |
| rs35431690 | *RBP4* | intronic | -- | 4 | -- | 6 tissues | ESC | Bbx,Pou2f2 | 1 hit |
| rs74599191 | *RBP4* | intronic | -- | 4 | -- | FAT | LNG | -- | 1 hit |
| rs1554753 | *RARG* | -- | TFBS | 4 | BLD | BLD | BLD | Evi-1 | 1 hit |
| rs2229774 | *RARG* | missense | nsSNP | 4 | -- | BLD, SKIN, LIV | BLD | 4 altered motifs | 1 hit |
| rs1465057 | *RARG* | intronic | -- | -- | 22 tissues | 7 tissues | 11 tissues | Smad3,ZBRK1 | 1 hit |
| rs941138 | *RARG* | intronic | -- | 3a | 23 tissues | BRST | 47 tissues | CEBPB,Pou2f2 | -- |
| rs6580936 | *RARG* | intronic | -- | 4 | 7 tissues | 21 tissues | 9 tissues | GR | 1 hit |
| rs3138139 | *RDH5* | intronic | -- | 3a | 4 tissues | 6 tissues | 6 tissues | ATF2,ATF4,E4BP4 | 6 hits |
| rs7134373 | *RDH16* | -- | -- | 4 | -- | -- | -- | ATF3 | 1 hit |
| rs901068 | *RDH16* | intronic | -- | 5 | -- | LIV | -- | -- | 3 hits |
| rs57077522 | *RDH12* | intronic | -- | 7 | -- | BLD | -- | Cdx,Zfp105 | 1 hit |
| rs8017326 | *RDH12* | intronic | -- | 4 | -- | LIV | -- | Nr2f2 | 5 hits |
| rs77718590 | *RDH12* | intronic | -- | 5 | LIV | 4 tissues | BRN,LNG | YY1 | -- |
| rs7169289 | *ALDH1A2* | -- | -- | 2b | -- | 4 tissues | BLD | Pou2f2,Smad,ZNF219 | -- |
| rs1441815 | *ALDH1A2* | intronic | TFBS | 5 | -- | -- | BLD | AP-1 | -- |
| rs12903202 | *ALDH1A2* | intronic | -- | 4 | -- | BLD | BLD | -- | 1 hit |
| rs10851633 | *ALDH1A2* | intronic | -- | 7 | -- | -- | -- | Sox | -- |
| rs6493977 | *ALDH1A2* | intronic | -- | 7 | -- | -- | -- | -- | 1 hit |
| rs8032107 | *ALDH1A2* | intronic | -- | 7 | -- | BRST, SKIN | -- | Pax-4,Pou2f2 | -- |
| rs10518961 | *ALDH1A2* | intronic | -- | 2c | -- | -- | BLD,VAS,BLD | 20 altered motifs | 1 hit |
| rs4646653 | *ALDH1A3* | intronic | -- | 7 | -- | -- | -- | Pou2f2 | -- |
| rs4646678 | *ALDH1A3* | intronic | -- | 4 | 12 tissues | 14 tissues | 15 tissues | -- | -- |
| rs9934274 | *BCMO1* | intronic | -- | 4 | -- | -- | -- | 5 altered motifs | -- |
| rs6564863 | *BCMO1* | intronic | -- | 2b | -- | GI | 24 tissues | 7 altered motifs | -- |
| rs7217852 | *RARA* | intronic | -- | 3a | STRM, GI, LNG | 22 tissues | 4 tissues | 4 altered motifs | -- |
| rs2715554 | *RARA* | intronic | -- | 1d | 9 tissues | 17 tissues | 5 tissues | Ik-3 | 28 hits |
| rs78340622 | *RDH8* | intronic | -- | -- | -- | SKIN | -- | 15 altered motifs | -- |
| rs1671216 | *RDH13* | -- | TFBS | 2b | BLD | 6 tissues | -- | -- | 34 hits |
| rs8113032 | *RDH13* | -- | TFBS | 1d | BLD | 6 tissues | -- | 6 altered motifs | 32 hits |
| rs1671218 | *RDH13* | -- | TFBS | 3a | -- | 5 tissues | PLCNT | HNF4,Roaz | 41 hits |
| rs62122050 | *RDH13* | -- | TFBS | 5 | -- | BLD, ADRL | -- | 5 altered motifs | 37 hits |
| rs4029 | *RDH13* | 3'-UTR | miRNA | 4 | -- | IPSC | LIV | SIX5 | 2 hits |
| rs775821 | *RDH13* | intronic | -- | 1f | -- | -- | IPSC,BLD | 7 altered motifs | 29 hits |
| rs34606618 | *RDH13* | intronic | -- | 5 | -- | -- | BLD | CEBPA,p300 | -- |
| rs55897280 | *RDH13* | intronic | -- | 4 | -- | -- | BLD | -- | 3 hits |
| rs1671169 | *RDH13* | intronic | -- | 1b | 22 tissues | 7 tissues | 10 tissues | RFX5 | 27 hits |
| rs1654466 | *RDH13* | intronic | -- | 4 | -- | 6 tissues | -- | -- | 4 hits |
| rs1654467 | *RDH13* | -- | TFBS | 4 | GI, CRVX | 9 tissues | ESDR,CRVX | -- | 2 hits |

**Supplementary Table 3. Association of 167 SNPs with prostate cancer risk**

| SNPs | Chr | Genes | Position | Allele^a^ | MAF | | *P*_(HWE)_^b^ | Call rate | Adjusted OR (95%CI) ^c^ | *P*^c^ | *P*_FDR_ |
| --- | --- | --- | --- | --- | --- | --- | --- | --- | --- | --- | --- |
|  |  |  |  |  | Cases | Controls |  |  |  |  |  |
| rs4075304 | 1 | *DHRS3* | 12626803 | T/C | 0.32 | 0.32 | 0.62 | 1.00 | 0.97 (0.91-1.04) | 5.05×10^-1^ | 0.933 |
| rs4072868 | 1 | *DHRS3* | 12628471 | C/A | 0.24 | 0.25 | 0.56 | 1.00 | 0.95 (0.88-1.02) | 2.22×10^-1^ | 0.933 |
| rs3128451 | 1 | *DHRS3* | 12641488 | G/T | 0.45 | 0.45 | 0.31 | 1.00 | 0.97 (0.91-1.04) | 5.25×10^-1^ | 0.933 |
| rs3128449 | 1 | *DHRS3* | 12641758 | G/A | 0.33 | 0.34 | 0.11 | 1.00 | 0.97 (0.91-1.04) | 4.63×10^-1^ | 0.933 |
| rs4846127 | 1 | *DHRS3* | 12663774 | C/T | 0.47 | 0.49 | 0.22 | 1.00 | 0.93 (0.87-0.99) | 4.44×10^-2^ | 0.933 |
| rs4394668 | 1 | *DHRS3* | 12671229 | C/T | 0.20 | 0.20 | 0.83 | 1.00 | 0.99 (0.91-1.07) | 8.60×10^-1^ | 0.933 |
| rs5022243 | 1 | *DHRS3* | 12671685 | G/C | 0.40 | 0.40 | 0.26 | 0.99 | 1.03 (0.97-1.10) | 2.53×10^-1^ | 0.933 |
| rs11121951 | 1 | *DHRS3* | 12676205 | T/C | 0.12 | 0.12 | 0.93 | 1.00 | 0.98 (0.89-1.08) | 7.71×10^-1^ | 0.933 |
| rs3125899 | 1 | *RPE65* | 68902237 | C/T | 0.39 | 0.39 | 0.08 | 1.00 | 0.99 (0.93-1.06) | 9.37×10^-1^ | 0.954 |
| rs17130694 | 1 | *RPE65* | 68909113 | T/C | 0.05 | 0.05 | 0.59 | 1.00 | 0.95 (0.82-1.10) | 5.68×10^-1^ | 0.933 |
| rs3790472 | 1 | *RPE65* | 68910999 | A/C | 0.47 | 0.46 | 0.66 | 0.99 | 1.04 (0.97-1.11) | 1.88×10^-1^ | 0.933 |
| rs283700 | 1 | *RXRG* | 165373146 | A/G | 0.13 | 0.14 | 0.25 | 0.99 | 0.97 (0.88-1.07) | 5.88×10^-1^ | 0.933 |
| rs10800096 | 1 | *RXRG* | 165376233 | G/C | 0.20 | 0.21 | 0.48 | 1.00 | 0.97 (0.89-1.05) | 5.26×10^-1^ | 0.933 |
| rs283694 | 1 | *RXRG* | 165376685 | T/C | 0.30 | 0.31 | 0.23 | 0.99 | 0.98 (0.92-1.05) | 7.02×10^-1^ | 0.933 |
| rs2651860 | 1 | *RXRG* | 165381055 | C/A | 0.20 | 0.21 | 0.11 | 1.00 | 0.98 (0.90-1.06) | 6.41×10^-1^ | 0.933 |
| rs12069160 | 1 | *RXRG* | 165381666 | G/T | 0.06 | 0.05 | 0.38 | 1.00 | 1.07 (0.93-1.23) | 3.23×10^-1^ | 0.933 |
| rs10489745 | 1 | *RXRG* | 165381844 | C/T | 0.09 | 0.08 | 0.90 | 0.99 | 1.05 (0.93-1.18) | 3.54×10^-1^ | 0.933 |
| rs188196 | 1 | *RXRG* | 165384621 | A/G | 0.15 | 0.15 | 0.45 | 1.00 | 0.96 (0.88-1.05) | 4.12×10^-1^ | 0.933 |
| rs6676763 | 1 | *RXRG* | 165387207 | A/G | 0.40 | 0.39 | 0.73 | 1.00 | 1.04 (0.97-1.11) | 2.43×10^-1^ | 0.933 |
| rs283691 | 1 | *RXRG* | 165387464 | A/G | 0.38 | 0.39 | 1.00 | 1.00 | 0.96 (0.90-1.02) | 2.43×10^-1^ | 0.933 |
| rs10918180 | 1 | *RXRG* | 165402460 | T/G | 0.10 | 0.10 | 1.00 | 1.00 | 0.96 (0.86-1.07) | 5.38×10^-1^ | 0.933 |
| rs74118603 | 1 | *RXRG* | 165406711 | A/T | 0.07 | 0.08 | 0.90 | 1.00 | 0.94 (0.83-1.06) | 3.55×10^-1^ | 0.933 |
| rs1831002 | 1 | *RXRG* | 165409735 | C/T | 0.15 | 0.15 | 0.78 | 0.99 | 1.00 (0.91-1.09) | 9.64×10^-1^ | 0.968 |
| rs752739 | 1 | *RXRG* | 165412959 | A/G | 0.23 | 0.24 | 0.24 | 1.00 | 0.97 (0.90-1.05) | 5.14×10^-1^ | 0.933 |
| rs1467664 | 1 | *RXRG* | 165414933 | C/T | 0.15 | 0.14 | 1.00 | 1.00 | 1.05 (0.95-1.15) | 2.91×10^-1^ | 0.933 |
| rs16985278 | 2 | *RDH14* | 18734072 | T/C | 0.26 | 0.25 | 0.78 | 1.00 | 1.03 (0.96-1.11) | 3.32×10^-1^ | 0.933 |
| rs13403096 | 2 | *RDH14* | 18734872 | C/T | 0.19 | 0.19 | 0.81 | 0.99 | 1.04 (0.95-1.12) | 3.57×10^-1^ | 0.933 |
| rs707718 | 2 | *CYP26B1* | 72356449 | T/G | 0.18 | 0.18 | 0.08 | 1.00 | 1.03 (0.95-1.12) | 3.71×10^-1^ | 0.933 |
| rs6753502 | 2 | *DHRS9* | 169943259 | A/G | 0.20 | 0.19 | 0.38 | 1.00 | 1.04 (0.96-1.13) | 2.98×10^-1^ | 0.933 |
| rs17532798 | 2 | *AOX1* | 201456390 | C/A | 0.15 | 0.15 | 0.33 | 1.00 | 1.01 (0.92-1.10) | 7.92×10^-1^ | 0.933 |
| rs1527945 | 2 | *AOX1* | 201456447 | T/C | 0.22 | 0.22 | 0.64 | 1.00 | 0.98 (0.91-1.07) | 7.96×10^-1^ | 0.933 |
| rs12993144 | 2 | *AOX1* | 201464565 | T/C | 0.40 | 0.40 | 0.02 | 1.00 | 0.98 (0.92-1.05) | 7.03×10^-1^ | 0.933 |
| rs13406379 | 2 | *AOX1* | 201469625 | A/G | 0.08 | 0.08 | 0.90 | 1.00 | 0.97 (0.86-1.10) | 6.94×10^-1^ | 0.933 |
| rs16833889 | 2 | *AOX1* | 201483925 | A/G | 0.35 | 0.35 | 0.03 | 1.00 | 0.98 (0.91-1.05) | 5.91×10^-1^ | 0.933 |
| rs59699466 | 2 | *AOX1* | 201485120 | T/C | 0.25 | 0.26 | 0.22 | 1.00 | 0.97 (0.90-1.04) | 4.54×10^-1^ | 0.933 |
| rs6732225 | 2 | *AOX1* | 201489552 | G/T | 0.33 | 0.34 | 0.02 | 1.00 | 0.97 (0.90-1.04) | 4.02×10^-1^ | 0.933 |
| rs6720189 | 2 | *AOX1* | 201489836 | C/T | 0.48 | 0.49 | 0.01 | 1.00 | 0.97 (0.91-1.04) | 5.24×10^-1^ | 0.933 |
| rs1986415 | 2 | *AOX1* | 201491560 | A/G | 0.11 | 0.11 | 1.00 | 1.00 | 0.99 (0.90-1.10) | 9.52×10^-1^ | 0.964 |
| rs2254576 | 2 | *AOX1* | 201496111 | A/G | 0.49 | 0.48 | 0.43 | 1.00 | 1.02 (0.95-1.08) | 5.45×10^-1^ | 0.933 |
| rs2465661 | 2 | *AOX1* | 201500631 | T/C | 0.35 | 0.36 | 0.56 | 1.00 | 0.98 (0.91-1.05) | 6.06×10^-1^ | 0.933 |
| rs7562507 | 2 | *AOX1* | 201523184 | G/C | 0.13 | 0.13 | 0.43 | 0.99 | 1.02 (0.93-1.12) | 5.87×10^-1^ | 0.933 |
| rs6435060 | 2 | *AOX1* | 201529900 | T/C | 0.39 | 0.40 | 0.74 | 1.00 | 0.99 (0.92-1.06) | 8.24×10^-1^ | 0.933 |
| rs4674311 | 2 | *AOX1* | 201531844 | C/T | 0.47 | 0.47 | 0.25 | 1.00 | 1.02 (0.95-1.09) | 4.77×10^-1^ | 0.933 |
| rs2715905 | 2 | *AOX1* | 201532625 | A/T | 0.37 | 0.38 | 0.49 | 1.00 | 0.97 (0.91-1.04) | 4.42×10^-1^ | 0.933 |
| rs2540066 | 2 | *AOX1* | 201535075 | A/G | 0.48 | 0.48 | 0.33 | 1.00 | 1.03 (0.97-1.10) | 2.77×10^-1^ | 0.933 |
| rs9825855 | 3 | *RARB* | 25268008 | G/C | 0.17 | 0.17 | 0.28 | 1.00 | 0.98 (0.89-1.06) | 6.48×10^-1^ | 0.933 |
| rs1483846 | 3 | *RARB* | 25319827 | C/G | 0.48 | 0.49 | 0.80 | 0.99 | 0.97 (0.91-1.04) | 4.53×10^-1^ | 0.933 |
| rs1436252 | 3 | *RARB* | 25335435 | G/A | 0.35 | 0.35 | 0.25 | 0.99 | 0.98 (0.92-1.05) | 6.79×10^-1^ | 0.933 |
| rs73154973 | 3 | *RARB* | 25336466 | C/T | 0.07 | 0.07 | 1.00 | 1.00 | 0.94 (0.82-1.06) | 3.34×10^-1^ | 0.933 |
| rs977226 | 3 | *RARB* | 25337483 | C/T | 0.20 | 0.19 | 1.00 | 0.99 | 1.03 (0.95-1.12) | 3.78×10^-1^ | 0.933 |
| rs2164478 | 3 | *RARB* | 25339647 | G/A | 0.44 | 0.43 | 0.71 | 0.99 | 1.02 (0.95-1.08) | 5.33×10^-1^ | 0.933 |
| rs1483836 | 3 | *RARB* | 25378658 | G/A | 0.38 | 0.38 | 0.14 | 0.99 | 0.99 (0.92-1.06) | 8.09×10^-1^ | 0.933 |
| rs74347859 | 3 | *RARB* | 25381352 | G/A | 0.11 | 0.11 | 0.03 | 0.99 | 0.97 (0.87-1.07) | 5.78×10^-1^ | 0.933 |
| rs1483830 | 3 | *RARB* | 25383713 | G/A | 0.40 | 0.40 | 0.01 | 0.99 | 0.96 (0.90-1.03) | 2.83×10^-1^ | 0.933 |
| rs1075410 | 3 | *RARB* | 25389853 | T/C | 0.28 | 0.29 | 0.03 | 1.00 | 0.96 (0.90-1.04) | 4.01×10^-1^ | 0.933 |
| rs1483843 | 3 | *RARB* | 25409842 | T/C | 0.20 | 0.20 | 0.53 | 1.00 | 1.04 (0.96-1.12) | 3.20×10^-1^ | 0.933 |
| rs13088742 | 3 | *RARB* | 25438508 | C/T | 0.17 | 0.16 | 0.95 | 1.00 | 1.01 (0.93-1.10) | 6.84×10^-1^ | 0.933 |
| rs2116701 | 3 | *RARB* | 25441977 | A/G | 0.15 | 0.14 | 0.18 | 1.00 | 1.04 (0.95-1.14) | 3.38×10^-1^ | 0.933 |
| rs59648542 | 3 | *RARB* | 25443467 | A/C | 0.13 | 0.13 | 0.87 | 0.99 | 1.01 (0.92-1.12) | 7.09×10^-1^ | 0.933 |
| rs6783726 | 3 | *RARB* | 25447603 | A/G | 0.31 | 0.31 | 0.21 | 0.99 | 1.00 (0.93-1.07) | 8.49×10^-1^ | 0.933 |
| rs4681060 | 3 | *RARB* | 25449650 | G/T | 0.12 | 0.13 | 0.42 | 0.99 | 0.96 (0.87-1.06) | 4.45×10^-1^ | 0.933 |
| rs6550974 | 3 | *RARB* | 25450943 | G/A | 0.40 | 0.40 | 0.08 | 1.00 | 1.01 (0.95-1.08) | 6.43×10^-1^ | 0.933 |
| rs9865116 | 3 | *RARB* | 25461807 | T/C | 0.08 | 0.08 | 0.72 | 1.00 | 1.02 (0.91-1.15) | 7.01×10^-1^ | 0.933 |
| rs6778608 | 3 | *RARB* | 25461836 | C/T | 0.22 | 0.22 | 0.09 | 0.99 | 1.00 (0.93-1.08) | 8.88×10^-1^ | 0.936 |
| rs755661 | 3 | *RARB* | 25472040 | T/C | 0.44 | 0.44 | 0.38 | 1.00 | 1.01 (0.94-1.07) | 7.24×10^-1^ | 0.933 |
| rs4681025 | 3 | *RARB* | 25475924 | G/A | 0.11 | 0.11 | 0.03 | 1.00 | 1.03 (0.93-1.14) | 5.26×10^-1^ | 0.933 |
| rs9284856 | 3 | *RARB* | 25479983 | A/G | 0.12 | 0.12 | 0.23 | 0.99 | 1.01 (0.91-1.11) | 8.34×10^-1^ | 0.933 |
| rs12631063 | 3 | *RARB* | 25485756 | G/A | 0.10 | 0.09 | 0.28 | 0.99 | 1.05 (0.94-1.18) | 3.28×10^-1^ | 0.933 |
| rs13314219 | 3 | *RARB* | 25495637 | C/A | 0.37 | 0.38 | 0.62 | 1.00 | 0.97 (0.91-1.04) | 5.01×10^-1^ | 0.933 |
| rs6778350 | 3 | *RARB* | 25507030 | A/G | 0.14 | 0.14 | 0.55 | 1.00 | 1.05 (0.96-1.15) | 2.72×10^-1^ | 0.933 |
| rs1529672 | 3 | *RARB* | 25520582 | A/C | 0.17 | 0.17 | 0.80 | 1.00 | 0.99 (0.90-1.07) | 8.20×10^-1^ | 0.933 |
| rs112050806 | 3 | *RARB* | 25538675 | T/C | 0.08 | 0.08 | 0.72 | 1.00 | 0.99 (0.87-1.11) | 8.70×10^-1^ | 0.933 |
| rs1881706 | 3 | *RARB* | 25550452 | A/G | 0.30 | 0.30 | 0.52 | 1.00 | 0.98 (0.91-1.05) | 6.84×10^-1^ | 0.933 |
| rs1153588 | 3 | *RARB* | 25550609 | C/G | 0.10 | 0.11 | 0.30 | 0.99 | 0.97 (0.87-1.07) | 5.76×10^-1^ | 0.933 |
| rs17526019 | 3 | *RARB* | 25574850 | T/C | 0.07 | 0.07 | 0.78 | 1.00 | 0.95 (0.84-1.08) | 4.62×10^-1^ | 0.933 |
| rs1286665 | 3 | *RARB* | 25575675 | T/C | 0.29 | 0.29 | 0.57 | 1.00 | 1.00 (0.93-1.08) | 8.32×10^-1^ | 0.933 |
| rs1881703 | 3 | *RARB* | 25578552 | A/C | 0.48 | 0.49 | 0.01 | 1.00 | 0.97 (0.91-1.03) | 3.52×10^-1^ | 0.933 |
| rs17016738 | 3 | *RARB* | 25600646 | T/C | 0.28 | 0.27 | 0.78 | 1.00 | 1.05 (0.98-1.13) | 1.26×10^-1^ | 0.933 |
| rs17016773 | 3 | *RARB* | 25604599 | T/C | 0.21 | 0.20 | 0.26 | 1.00 | 1.08 (1.00-1.17) | 4.77×10^-2^ | 0.933 |
| rs77132200 | 3 | *RARB* | 25608843 | G/T | 0.07 | 0.07 | 1.00 | 0.99 | 0.96 (0.84-1.09) | 5.49×10^-1^ | 0.933 |
| rs1286733 | 3 | *RARB* | 25613372 | C/T | 0.39 | 0.39 | 0.36 | 1.00 | 1.01 (0.94-1.08) | 7.30×10^-1^ | 0.933 |
| rs1286734 | 3 | *RARB* | 25613889 | C/A | 0.12 | 0.11 | 0.64 | 1.00 | 1.09 (0.98-1.21) | 8.22×10^-2^ | 0.933 |
| rs1286738 | 3 | *RARB* | 25614532 | T/C | 0.22 | 0.21 | 0.74 | 1.00 | 1.06 (0.98-1.15) | 1.15×10^-1^ | 0.933 |
| rs4681028 | 3 | *RARB* | 25628749 | T/G | 0.21 | 0.20 | 0.42 | 1.00 | 1.09 (1.00-1.18) | 2.92×10^-2^ | 0.933 |
| rs7621140 | 3 | *RARB* | 25630857 | C/T | 0.12 | 0.11 | 0.07 | 1.00 | 1.06 (0.96-1.18) | 2.13×10^-1^ | 0.933 |
| rs1058378 | 3 | *RARB* | 25639394 | G/T | 0.10 | 0.09 | 0.19 | 1.00 | 1.07 (0.96-1.20) | 1.92×10^-1^ | 0.933 |
| rs2032348 | 4 | *ADH4* | 100064247 | T/G | 0.13 | 0.13 | 1.00 | 1.00 | 0.98 (0.89-1.07) | 7.01×10^-1^ | 0.933 |
| rs1800761 | 4 | *ADH4* | 100065593 | T/C | 0.19 | 0.19 | 0.49 | 1.00 | 1.01 (0.93-1.09) | 8.07×10^-1^ | 0.933 |
| rs4148884 | 4 | *ADH4* | 100066287 | T/C | 0.08 | 0.08 | 1.00 | 1.00 | 1.01 (0.90-1.14) | 8.05×10^-1^ | 0.933 |
| rs2851012 | 4 | *ADH7* | 100331956 | C/T | 0.39 | 0.40 | 0.47 | 0.99 | 0.97 (0.91-1.04) | 5.24×10^-1^ | 0.933 |
| rs2584463 | 4 | *ADH7* | 100332697 | T/G | 0.20 | 0.19 | 1.00 | 1.00 | 1.03 (0.95-1.12) | 3.64×10^-1^ | 0.933 |
| rs284786 | 4 | *ADH7* | 100333977 | A/T | 0.30 | 0.30 | 0.73 | 0.99 | 0.99 (0.92-1.06) | 8.46×10^-1^ | 0.933 |
| rs4147551 | 4 | *ADH7* | 100335907 | A/C | 0.07 | 0.06 | 1.00 | 1.00 | 1.03 (0.90-1.17) | 6.61×10^-1^ | 0.933 |
| rs1827567 | 4 | *ADH7* | 100336102 | A/G | 0.23 | 0.23 | 0.26 | 0.99 | 1.00 (0.93-1.08) | 8.48×10^-1^ | 0.933 |
| rs1154456 | 4 | *ADH7* | 100339596 | G/A | 0.33 | 0.32 | 0.84 | 1.00 | 1.02 (0.96-1.10) | 4.30×10^-1^ | 0.933 |
| rs1154458 | 4 | *ADH7* | 100340522 | C/G | 0.41 | 0.41 | 0.91 | 0.99 | 0.99 (0.93-1.06) | 9.37×10^-1^ | 0.954 |
| rs1154460 | 4 | *ADH7* | 100341643 | A/G | 0.45 | 0.45 | 0.69 | 1.00 | 1.00 (0.94-1.07) | 8.75×10^-1^ | 0.933 |
| rs201824 | 4 | *LRAT* | 155668710 | A/G | 0.36 | 0.36 | 0.53 | 1.00 | 0.97 (0.91-1.04) | 5.50×10^-1^ | 0.933 |
| rs78274322 | 4 | *LRAT* | 155671105 | G/A | 0.08 | 0.09 | 0.50 | 1.00 | 0.93 (0.83-1.05) | 2.91×10^-1^ | 0.933 |
| rs1546877 | 6 | *RXRB* | 33161262 | C/T | 0.32 | 0.32 | 1.00 | 0.99 | 1.00 (0.93-1.07) | 8.91×10^-1^ | 0.936 |
| rs2076310 | 6 | *RXRB* | 33166034 | G/A | 0.23 | 0.23 | 0.69 | 1.00 | 0.97 (0.90-1.05) | 5.40×10^-1^ | 0.933 |
| rs13279861 | 8 | *SDR16C5* | 57211120 | G/A | 0.35 | 0.35 | 0.94 | 0.99 | 1.01 (0.94-1.08) | 7.31×10^-1^ | 0.933 |
| rs4419802 | 8 | *SDR16C5* | 57216066 | A/G | 0.28 | 0.27 | 0.93 | 1.00 | 1.04 (0.97-1.12) | 2.38×10^-1^ | 0.933 |
| rs7819550 | 8 | *RDH10* | 74206582 | A/G | 0.19 | 0.18 | 0.47 | 1.00 | 1.04 (0.96-1.13) | 3.09×10^-1^ | 0.933 |
| rs35680921 | 8 | *RDH10* | 74208422 | A/G | 0.47 | 0.47 | 0.13 | 0.99 | 0.98 (0.92-1.04) | 6.25×10^-1^ | 0.933 |
| rs4737370 | 8 | *RDH10* | 74212259 | C/T | 0.26 | 0.26 | 0.01 | 0.99 | 1.00 (0.93-1.08) | 8.67×10^-1^ | 0.933 |
| rs2925455 | 8 | *RDH10* | 74217659 | C/A | 0.06 | 0.07 | 0.11 | 1.00 | 0.97 (0.85-1.11) | 7.23×10^-1^ | 0.933 |
| rs4738319 | 8 | *RDH10* | 74228064 | T/C | 0.23 | 0.22 | 0.35 | 0.99 | 1.03 (0.95-1.11) | 3.77×10^-1^ | 0.933 |
| rs17215061 | 8 | *RDH10* | 74234437 | A/C | 0.21 | 0.22 | 0.26 | 0.99 | 0.98 (0.90-1.06) | 6.70×10^-1^ | 0.933 |
| rs348482 | 9 | *ALDH1A1* | 75514717 | A/G | 0.23 | 0.23 | 0.54 | 1.00 | 1.02 (0.94-1.10) | 5.58×10^-1^ | 0.933 |
| rs168351 | 9 | *ALDH1A1* | 75517311 | G/A | 0.16 | 0.16 | 0.32 | 0.99 | 0.98 (0.90-1.07) | 7.26×10^-1^ | 0.933 |
| rs4646547 | 9 | *ALDH1A1* | 75517582 | C/A | 0.50 | 0.49 | 0.14 | 1.00 | 1.01 (0.95-1.08) | 6.09×10^-1^ | 0.933 |
| rs348461 | 9 | *ALDH1A1* | 75545070 | T/A | 0.36 | 0.36 | 0.67 | 1.00 | 0.97 (0.91-1.04) | 5.33×10^-1^ | 0.933 |
| rs13959 | 9 | *ALDH1A1* | 75545882 | G/A | 0.49 | 0.50 | 1.00 | 1.00 | 0.97 (0.91-1.03) | 4.20×10^-1^ | 0.933 |
| rs1330286 | 9 | *ALDH1A1* | 75552953 | G/C | 0.33 | 0.36 | 0.01 | 1.00 | 0.88 (0.83-0.94) | 2.45×10^-4^ | 0.036 |
| rs10869206 | 9 | *ALDH1A1* | 75593971 | C/T | 0.31 | 0.32 | 0.48 | 1.00 | 0.96 (0.90-1.03) | 3.83×10^-1^ | 0.933 |
| rs62560451 | 9 | *ALDH1A1* | 75597138 | A/G | 0.25 | 0.26 | 0.51 | 0.99 | 0.96 (0.90-1.04) | 4.02×10^-1^ | 0.933 |
| rs7860374 | 9 | *ALDH1A1* | 75633586 | G/A | 0.26 | 0.26 | 0.93 | 1.00 | 0.97 (0.90-1.04) | 4.87×10^-1^ | 0.933 |
| rs2310308 | 9 | *ALDH1A1* | 75634620 | A/G | 0.33 | 0.34 | 0.78 | 0.99 | 0.96 (0.89-1.02) | 2.43×10^-1^ | 0.933 |
| rs11102986 | 9 | *RXRA* | 137285503 | A/G | 0.18 | 0.18 | 0.00 | 1.00 | 0.98 (0.90-1.07) | 7.66×10^-1^ | 0.933 |
| rs12115573 | 9 | *RXRA* | 137288782 | G/A | 0.37 | 0.37 | 0.03 | 0.99 | 0.99 (0.92-1.05) | 7.74×10^-1^ | 0.933 |
| rs12004589 | 9 | *RXRA* | 137290725 | T/G | 0.13 | 0.13 | 0.42 | 0.99 | 0.99 (0.89-1.09) | 8.52×10^-1^ | 0.933 |
| rs3118529 | 9 | *RXRA* | 137304915 | C/T | 0.32 | 0.33 | 0.06 | 1.00 | 0.98 (0.91-1.05) | 6.02×10^-1^ | 0.933 |
| rs877954 | 9 | *RXRA* | 137326408 | A/G | 0.35 | 0.35 | 0.61 | 1.00 | 0.98 (0.91-1.05) | 6.05×10^-1^ | 0.933 |
| rs1538648 | 10 | *CYP26C1* | 94820860 | A/G | 0.50 | 0.49 | 0.22 | 0.99 | 1.03 (0.97-1.10) | 2.88×10^-1^ | 0.933 |
| rs4919592 | 10 | *CYP26C1* | 94823413 | A/G | 0.09 | 0.09 | 0.16 | 0.99 | 0.96 (0.86-1.07) | 5.22×10^-1^ | 0.933 |
| rs4411227 | 10 | *CYP26A1* | 94831513 | G/C | 0.26 | 0.27 | 0.75 | 0.99 | 0.97 (0.90-1.05) | 5.20×10^-1^ | 0.933 |
| rs11187541 | 10 | *RBP4* | 95351059 | C/T | 0.13 | 0.12 | 0.62 | 0.99 | 1.02 (0.93-1.13) | 6.01×10^-1^ | 0.933 |
| rs7094671 | 10 | *RBP4* | 95355617 | A/G | 0.24 | 0.24 | 0.49 | 1.00 | 1.04 (0.97-1.12) | 2.36×10^-1^ | 0.933 |
| rs35431690 | 10 | *RBP4* | 95356132 | T/C | 0.16 | 0.16 | 0.69 | 1.00 | 1.02 (0.94-1.12) | 5.31×10^-1^ | 0.933 |
| rs74599191 | 10 | *RBP4* | 95357753 | A/G | 0.08 | 0.08 | 1.00 | 1.00 | 1.06 (0.94-1.20) | 2.80×10^-1^ | 0.933 |
| rs1554753 | 12 | *RARG* | 53603738 | G/A | 0.19 | 0.19 | 0.32 | 1.00 | 1.01 (0.93-1.10) | 7.44×10^-1^ | 0.933 |
| rs2229774 | 12 | *RARG* | 53605545 | A/G | 0.07 | 0.07 | 0.66 | 1.00 | 1.00 (0.88-1.14) | 9.68×10^-1^ | 0.968 |
| rs1465057 | 12 | *RARG* | 53612881 | C/T | 0.09 | 0.08 | 0.20 | 1.00 | 1.01 (0.90-1.14) | 7.47×10^-1^ | 0.933 |
| rs941138 | 12 | *RARG* | 53614349 | C/T | 0.07 | 0.07 | 0.21 | 1.00 | 1.04 (0.91-1.18) | 5.16×10^-1^ | 0.933 |
| rs6580936 | 12 | *RARG* | 53617829 | G/A | 0.16 | 0.16 | 0.10 | 1.00 | 0.99 (0.90-1.08) | 8.44×10^-1^ | 0.933 |
| rs3138139 | 12 | *RDH5* | 56115883 | C/T | 0.09 | 0.09 | 0.10 | 1.00 | 1.04 (0.92-1.17) | 4.82×10^-1^ | 0.933 |
| rs7134373 | 12 | *RDH16* | 57343877 | C/G | 0.09 | 0.08 | 0.30 | 1.00 | 1.01 (0.91-1.14) | 7.40×10^-1^ | 0.933 |
| rs901068 | 12 | *RDH16* | 57346805 | G/T | 0.09 | 0.09 | 0.75 | 1.00 | 0.91 (0.82-1.02) | 1.40×10^-1^ | 0.933 |
| rs57077522 | 14 | *RDH11* | 68169527 | C/T | 0.08 | 0.09 | 0.74 | 1.00 | 0.93 (0.83-1.04) | 2.42×10^-1^ | 0.933 |
| rs8017326 | 14 | *RDH12* | 68183399 | A/G | 0.14 | 0.13 | 0.04 | 0.99 | 1.06 (0.97-1.17) | 1.68×10^-1^ | 0.933 |
| rs77718590 | 14 | *RDH12* | 68197012 | G/A | 0.10 | 0.11 | 0.10 | 1.00 | 0.96 (0.86-1.06) | 4.70×10^-1^ | 0.933 |
| rs7169289 | 15 | *ALDH1A2* | 58243683 | G/A | 0.19 | 0.18 | 0.81 | 0.99 | 1.01 (0.93-1.10) | 7.04×10^-1^ | 0.933 |
| rs1441815 | 15 | *ALDH1A2* | 58274229 | A/C | 0.43 | 0.43 | 0.29 | 1.00 | 0.96 (0.90-1.02) | 2.56×10^-1^ | 0.933 |
| rs12903202 | 15 | *ALDH1A2* | 58306793 | G/A | 0.09 | 0.09 | 0.12 | 1.00 | 0.97 (0.87-1.08) | 6.11×10^-1^ | 0.933 |
| rs10851633 | 15 | *ALDH1A2* | 58323065 | T/C | 0.37 | 0.36 | 0.28 | 1.00 | 1.04 (0.97-1.11) | 1.92×10^-1^ | 0.933 |
| rs6493977 | 15 | *ALDH1A2* | 58325944 | T/C | 0.46 | 0.47 | 0.25 | 1.00 | 0.97 (0.91-1.04) | 4.39×10^-1^ | 0.933 |
| rs8032107 | 15 | *ALDH1A2* | 58326834 | G/C | 0.48 | 0.48 | 0.54 | 0.99 | 1.02 (0.95-1.09) | 5.00×10^-1^ | 0.933 |
| rs10518961 | 15 | *ALDH1A2* | 58334345 | A/G | 0.45 | 0.44 | 0.34 | 1.00 | 1.05 (0.98-1.12) | 1.37×10^-1^ | 0.933 |
| rs4646653 | 15 | *ALDH1A3* | 101424476 | C/T | 0.18 | 0.16 | 0.31 | 0.99 | 1.17 (1.07-1.27) | 4.33×10^-4^ | 0.036 |
| rs4646678 | 15 | *ALDH1A3* | 101442436 | T/C | 0.19 | 0.17 | 0.18 | 1.00 | 1.14 (1.05-1.24) | 1.33×10^-3^ | 0.074 |
| rs9934274 | 16 | *BCO1* | 81283829 | G/C | 0.42 | 0.41 | 0.08 | 0.99 | 1.01 (0.94-1.08) | 7.15×10^-1^ | 0.933 |
| rs6564863 | 16 | *BCO1* | 81294893 | T/C | 0.34 | 0.34 | 0.63 | 1.00 | 0.96 (0.90-1.03) | 3.67×10^-1^ | 0.933 |
| rs7217852 | 17 | *RXRB* | 38470021 | G/A | 0.13 | 0.12 | 0.28 | 0.99 | 1.06 (0.96-1.17) | 2.32×10^-1^ | 0.933 |
| rs2715554 | 17 | *RXRB* | 38489170 | G/A | 0.14 | 0.14 | 0.16 | 1.00 | 0.99 (0.90-1.08) | 8.61×10^-1^ | 0.933 |
| rs78340622 | 19 | *RDH8* | 10128936 | A/T | 0.46 | 0.47 | 0.35 | 0.99 | 0.95 (0.89-1.01) | 1.55×10^-1^ | 0.933 |
| rs1671216 | 19 | *RDH13* | 55554076 | G/A | 0.38 | 0.38 | 0.14 | 0.99 | 1.01 (0.94-1.08) | 7.37×10^-1^ | 0.933 |
| rs8113032 | 19 | *RDH13* | 55554138 | A/G | 0.42 | 0.42 | 0.27 | 0.99 | 1.01 (0.95-1.08) | 6.70×10^-1^ | 0.933 |
| rs1671218 | 19 | *RDH13* | 55554399 | C/T | 0.34 | 0.34 | 0.58 | 1.00 | 1.00 (0.93-1.07) | 9.22×10^-1^ | 0.951 |
| rs62122050 | 19 | *RDH13* | 55554550 | C/A | 0.15 | 0.15 | 0.28 | 0.99 | 1.02 (0.93-1.12) | 5.53×10^-1^ | 0.933 |
| rs4029 | 19 | *RDH13* | 55555845 | G/A | 0.14 | 0.14 | 0.56 | 1.00 | 0.95 (0.87-1.04) | 3.18×10^-1^ | 0.933 |
| rs775821 | 19 | *RDH13* | 55558568 | A/G | 0.29 | 0.29 | 0.52 | 1.00 | 0.99 (0.92-1.06) | 8.77×10^-1^ | 0.933 |
| rs34606618 | 19 | *RDH13* | 55563959 | T/C | 0.07 | 0.07 | 0.40 | 1.00 | 0.98 (0.86-1.12) | 8.61×10^-1^ | 0.933 |
| rs55897280 | 19 | *RDH13* | 55568438 | T/C | 0.10 | 0.11 | 0.71 | 0.99 | 0.96 (0.86-1.07) | 4.83×10^-1^ | 0.933 |
| rs1671169 | 19 | *RDH13* | 55573633 | A/G | 0.25 | 0.25 | 0.16 | 0.99 | 1.00 (0.93-1.08) | 9.16×10^-1^ | 0.950 |
| rs1654466 | 19 | *RDH13* | 55578702 | G/A | 0.42 | 0.42 | 0.74 | 1.00 | 0.99 (0.93-1.06) | 9.05×10^-1^ | 0.945 |
| rs1654467 | 19 | *RDH13* | 55581511 | C/T | 0.40 | 0.39 | 0.29 | 1.00 | 1.04 (0.97-1.11) | 2.02×10^-1^ | 0.933 |

Abbreviations: OR odds ratio, CI confidence interval, HWE: Hardy-Weinberg Equilibrium

^a^ Reference allele/effect allele

^b^ *P* for additive model adjusted for age and smoking status in logistic regression model

^c^ *P* after false discovery rate correction

**Supplementary Figure legends**

**Supplementary Fig.1** **Selected genes in retinol metabolism pathway.** A brief diagram of 31 genes selected in this study and the metabolism of retinol.

Abbreviations: 9cROL, 9-cis-retinol; 9cRAL, 9-cis-retinal;9cRA, 9-cis-retinoic acid; atROL, all-trans-retinol; atRAL, all-trans-retinal; atRA, all-trans-retinoic acid; atRE, all-trans-retinyl esters; 11cROL, 11-cis-retinol; 11cRAL, 11-cis-retinal; RBP4, retinol binding protein 4;

**Supplementary Fig.2 The eQTL analysis of** **rs1330286 in *ALDH1A1* in prostate tissue samples in GTEx database.**

NES: Normalized effect size, defined as the slope of the linear regression computed in a normalized space. *P*-value: from a *t*-test that compares observed beta from single-tissue eQTL analysis to a null beta of 0.

**Supplementary Fig.1**


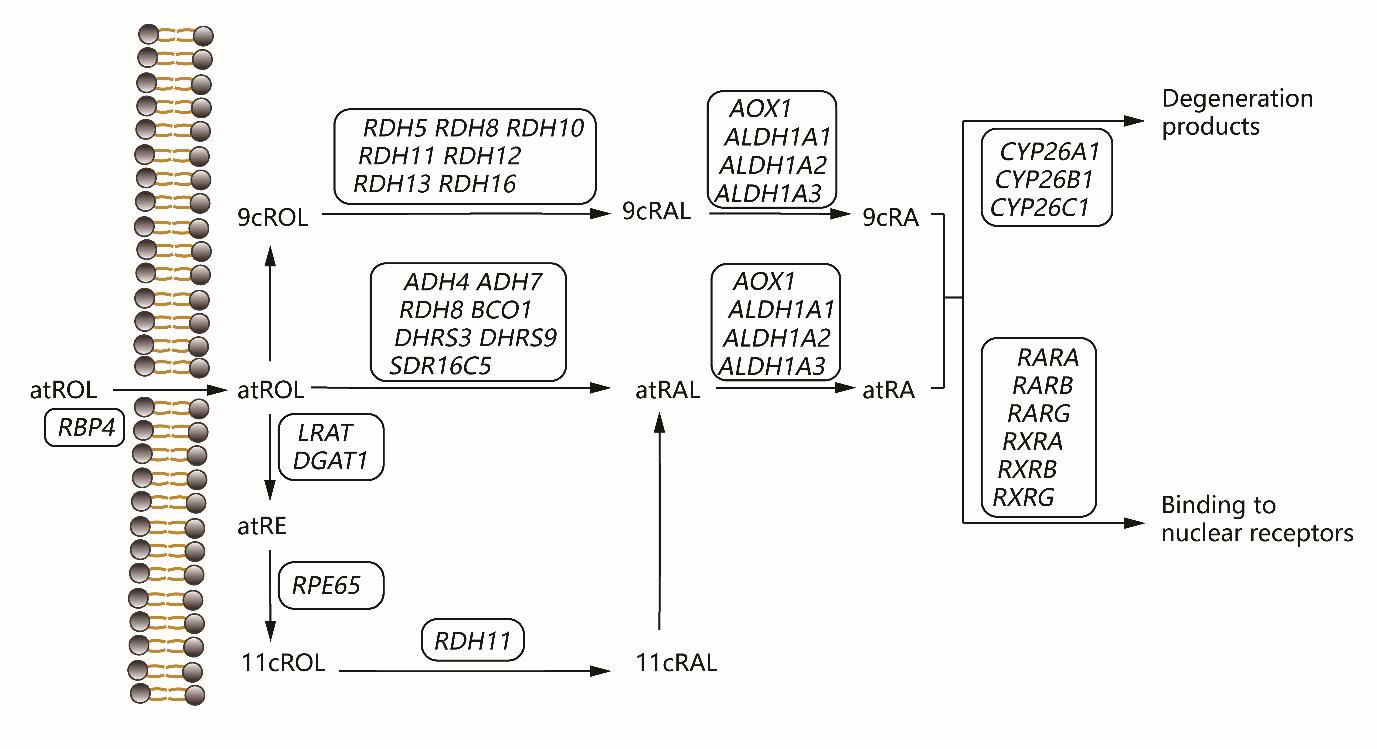


**Supplementary Fig.2**


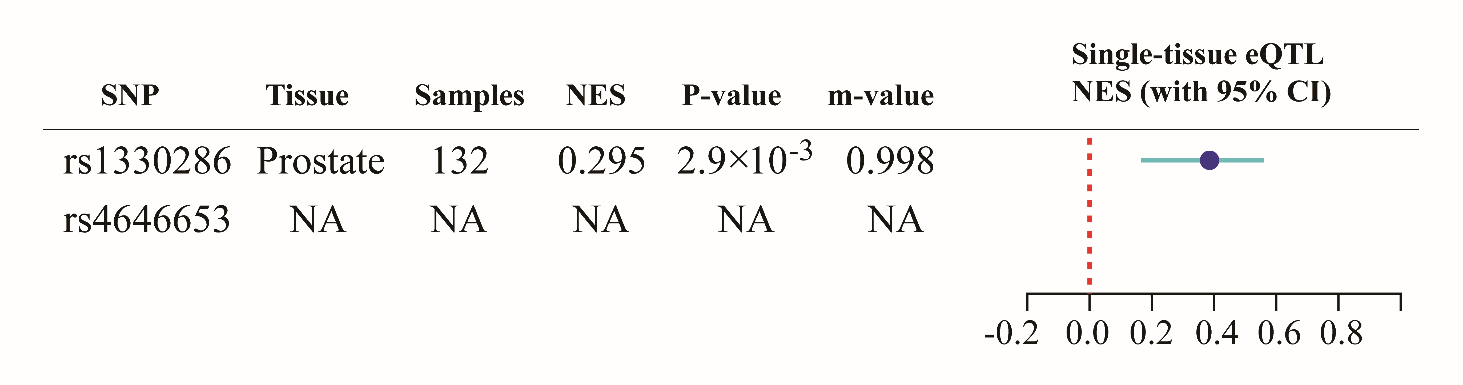


NA: Not Available.
